# Same FeN4 Active Site, Different Activity: How Redox Peaks Control Oxygen Reduction on Fe Macrocycles

**DOI:** 10.1021/acselectrochem.4c00146

**Published:** 2025-01-08

**Authors:** Silvia Favero, Ruixuan Chen, Joyce Cheung, Luke Higgins, Hui Luo, Mengnan Wang, Jesus Barrio, Maria Magdalena Titirici, Alexander Bagger, Ifan E. L. Stephens

**Affiliations:** † Department of Chemical Engineering, Imperial College London, South Kensington Campus, SW7 2AZ London, United Kingdom; ‡ Diamond Light Source, Didcot OX11 0DE, United Kingdom; § Institute for Sustainability, University of Surrey, Surrey GU2 7XH, United Kingdom; ∥ Department of Physics, Danish Technical University, 2800 Kongens Lyngby, Denmark; ⊥ Department of Materials, Imperial College London, South Kensington Campus, SW7 2AZ London, United Kingdom

**Keywords:** Oxygen reduction mechanism, Macrocycles, Fe−N−C electrocatalysts, CV redox peaks

## Abstract

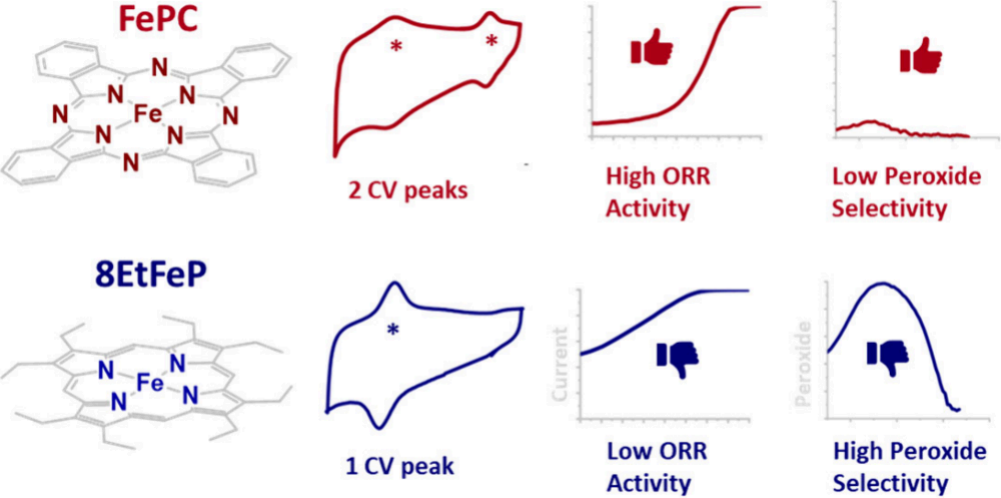

Macrocycles show high activity for the electrochemical reduction of oxygen in alkaline media. However, even macrocycles with the same metal centers and MN4 active site can vary significantly in activity and selectivity, and to this date, a quantitative insight into the cause of these staggering differences has not been unambiguously reached. These macrocycles form a fundamental platform, similarly to platinum alloys for metal ORR catalyst, to unravel fundamental properties of FeNx catalysts. In this manuscript, we present a systematic study of several macrocycles, with varying active site motif and ligands, using electrochemical techniques, *operando* spectroscopy, and density functional theory (DFT) simulations. Our study demonstrates the existence of two families of Fe macrocycles for oxygen reduction in alkaline electrolytes: (i) weak *OH binding macrocycles with one peak in the voltammogram and high peroxide selectivity and (ii) macrocycles with close to optimal *OH binding, which exhibit two voltametric peaks and almost no peroxide production. Here, we also propose three mechanisms that would explain our experimental findings. Understanding what differentiates these two families could shed light on how to optimize the activity of pyrolyzed FeNx catalysts.

## Introduction

The energy crisis and the threat of global warming have fueled research into carbon-neutral energy sources. Hydrogen fuel cells appear as an attractive option to transform chemical energy into clean electricity. Low temperature fuel cells can be divided into two categories: those operating in acidic pH, proton exchange membrane fuel cells, and those operating in alkaline conditions, anion exchange membrane fuel cells. Independently of pH, the oxygen reduction reaction (ORR) taking place at the cathode of fuel cells is the main source of inefficiency in these devices. The reason behind this inefficiency is that oxygen reduction, involving four electron and four proton transfers, with several reaction intermediates, requires a high overpotential. The experimental elucidation of the reaction mechanism is challenging because the intermediates cannot be easily probed *in situ*. Density functional theory calculations suggest that oxygen reduction proceeds through three key intermediates: *OOH, *O, and *OH.^1^ To allow the reaction to take place at low overpotentials, one should be able to independently control the binding energy of each intermediate in a way to minimize the energy requirement of each step. For the case of Pt(111), the potential-determining steps are the formation of the HOO* intermediate (step 1) and the reduction of the HO* intermediate (step 4), and therefore, activity could be improved by strengthening the *OOH bond and weakening the *OH bond. However, it is not possible to independently control the adsorption energy of these intermediates, as they scale linearly.^2,3^ This observation gives rise to a volcano-type dependence of the ORR activity on the binding energy of any oxygenated intermediate and to a theoretical minimum overpotential of ca. 0.4 eV.^4,5^ Additionally, the state-of-the-art catalysts are based on precious metals, which prohibitive costs and scarcity could limit widespread commercialization of fuel cells. Thanks to recent progresses in the anion-exchange membranes, alkaline fuel cells have received renewed attention.^6^ The alkaline AEMFC holds additional advantages over its acidic counterpart, such as improved material stability, easier water management, and improved ORR kinetics for noble metal-free catalysts. From the extensive research into noble metal-free catalysts for oxygen reduction, metal–nitrogen–carbon (M–N–C) materials have stood out for their high activity, especially in alkaline media. Noble metal-free M–N–C catalysts are composed of earth abundant transition metals (M), such as Fe, Co, Mn, and Ni, atomically dispersed in a nitrogen-rich carbon matrix.^7,8^ Most commonly, these catalysts are prepared from the pyrolysis of a variety of carbon and nitrogen precursors, along with metal salts.^9^ The pyrolysis step is responsible for an increase in both activity and durability of M–N–C catalysts but also leads to the formation of a heterogeneous structure, often containing, on top of the desired atomically dispersed nitrogen-coordinated metal sites (MN_*x*_), metal carbides, metallic nanoparticles, and metal oxides.^10−13^ Advanced characterization techniques, such as such X-ray absorption fine structure (XAFS),^14^ electron energy loss spectroscopy, and Mossbauer spectroscopy,^15−17^ have been applied in the attempt to further understand the coordination environment and electronic state of the metal active center. FeN_4_ groups are generally recognized as the active site,^18^ but the heterogeneity of pyrolyzed catalysts and the difficulty still experienced in characterizing the iron sites make it challenging to draw a structure-performance relationship and to rationally improve the design of FeN_4_ catalysts.

Macrocycles, on the other hand, offer well-defined and unique MN_4_ metal centers, allowing for an easier study of the active site for oxygen reduction in these MNC materials.^19^ Despite their high activity, macrocycles have not been studied for practical fuel cell applications due to their limited stability, particularly in acidic conditions.^20,21^ To overcome this limitation, some strategies have emerged, which improve the stability of macrocycles and often increase the activity via an electron-withdrawing effect at the metal center.^22^ These strategies include penta-coordination,^23−27^ by which the macromolecules are bound to a support by an axial ligand and pyrolysis,^25,28−30^ which improves activity and stability but leads to the progressive loss of the well-defined structure. Nevertheless, the unmodified macrocycles offer a great platform for fundamental mechanistic and kinetic studies, as their exact structural motif can be directly used in DFT simulation to gain a one-to-one correspondence with experimental results and quantum chemistry simulations.^31−36^ Several Fe macrocycles are commercially available, featuring porphyrin and phthalocyanine motifs, as well as various substituents. This allows one to modify the electron density on the metal center and observe its effect on the interaction with oxygenated ORR intermediates and on the catalytic properties of the active sites.^34,37−43^

For Fe macrocycles, an interesting feature is that they exhibit peaks in their cyclic voltammograms (CVs) under nitrogen in both acidic and alkaline media (over a typical potential range of 0 to 1 V vs RHE). Most macrocycles present two CV peaks, and the general consensus assigns the peak positioned at the highest potential to the desorption of the hydroxyl intermediate (*OH) and concomitant reduction of the metal center from Fe^3+^ to Fe^2+^, while the peak at a lower potential has been attributed to a further reduction of the metal center to Fe^1+^ ^44,45^ or less frequently to the reduction of the cycle surrounding the metal center.^46^ Other macrocycles, typically Fe porphyrins, only exhibit one CV peak in a potential range of 0–1 V vs RHE, which is commonly attributed to *OH desorption.^45^ The understanding of the CV peaks is of fundamental importance: if the peak position is due to the adsorption/desorption of *OH, then the potential should be an experimental measure of the *OH binding energy and, via the scaling relationship between reaction intermediates, a descriptor of the ORR activity via a Sabatier volcano plot.^47^

Indeed, Zagal and Koper established that there is a correlation between the peaks in the CV and the oxygen reduction activity for a relatively large amount of macrocycles.^45^ Nonetheless, some inconsistencies remain unexplained. For catalysts that mainly promote the 4-electron mechanism, they proposed that *OH acts as a poison for the metal and that M^3+^–OH is an inactive form of the MN_4_ catalysts.^48^ Therefore, *OH desorption should be the first step of the catalytic cycle, and the overpotential for oxygen reduction at low current density should closely match the CV peak. However, in many cases, oxygen reduction current can be observed at potentials up to 500 mV positive of the CV peak, where the catalyst should putatively be inactive due to the strongly bound *OH. On the other hand, most pyrolyzed catalysts do not present any CV peak. This is most often explained by assuming that these peaks are present but not visible, due to either the existence of a broad range of Fe sites and an equally broad collection *OH desorption potential, or the high capacitance of the carbon support.^49,50^

To address these inconsistencies and elucidate what controls the activity of single atom catalysts, this article will make use of a library of Fe macrocycles. We will focus on iron as the metal center, since FeN_4_ has been predicted to be the most active transition metal site for oxygen reduction,^38^ and consequently, Fe–N–C is also the most studied catalyst in the literature. We will use a combination of experimental techniques, in situ characterization, and DFT predictions to elucidate the nature of the CV peaks. We will then investigate the reason why some macromolecules only exhibit one peak, and we will correlate these data to their ORR activity.

## Results

### Accurate Identification of Voltammetric Peaks

**Figure 1:**
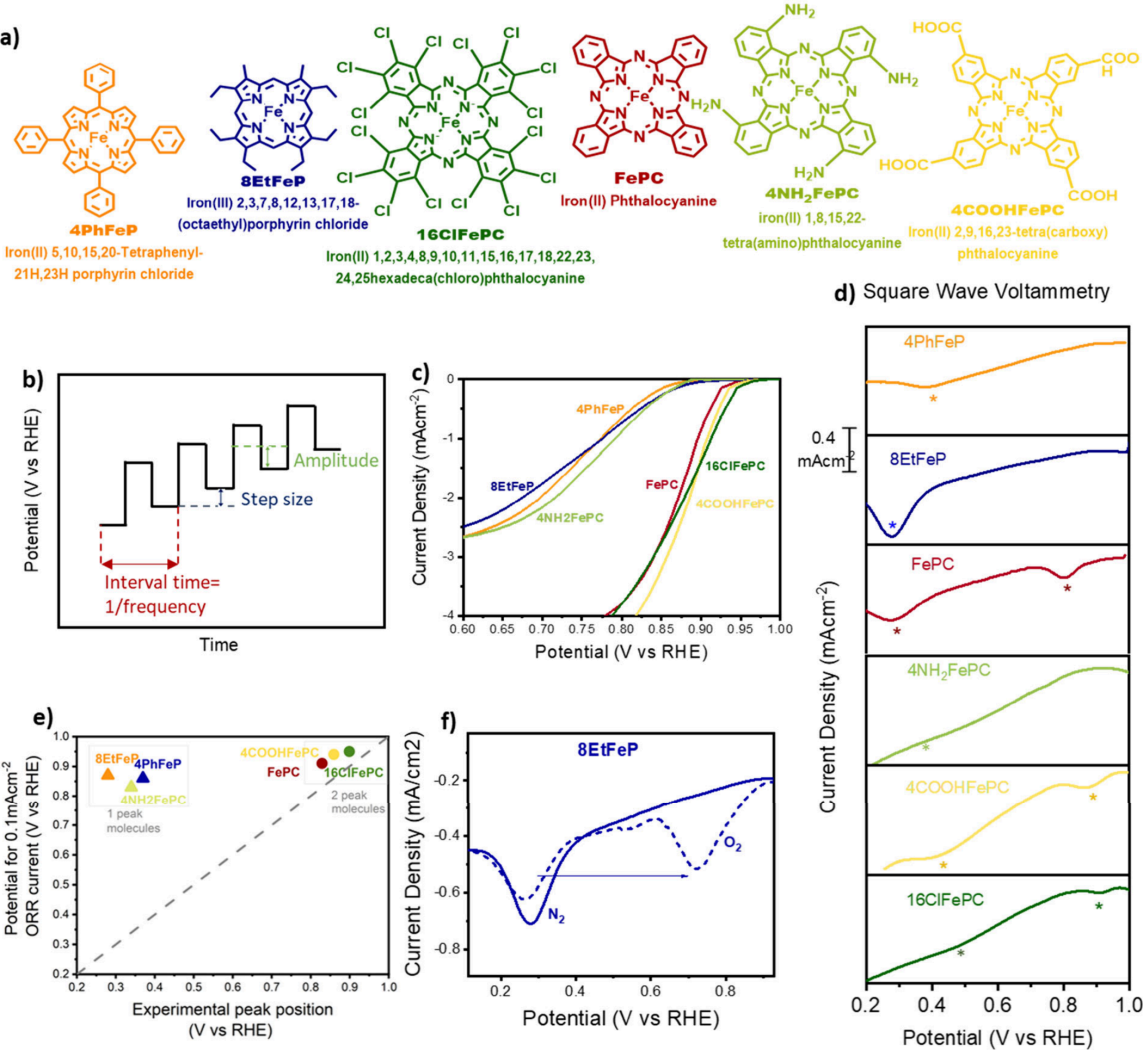
(a) Structure and abbreviation of Fe macromolecules used in this work. (b) Schematic representation of a square wave voltammogram. (c) Linear sweep voltammogram of the selected Fe macrocycles, obtained in oxygen-saturated 0.1 M KOH, at a scan rate of 10 mVs^–1^. (d) Square wave voltammograms of 4PhFeP (orange), 8EtFeP (blue), FePC (red), 4NH_2_FePC (light green), 4COOHFePC (yellow), and 16ClFePC (green). Data were collected in N_2_-saturated 0.1 M KOH, in static conditions, using a cathodic scan composed of a potential step of 4 mV, modulation amplitude of 20 mV, and a frequency of 2 Hz, resulting in a scan rate of 8 mVs^–1^. The peak position is highlighted by an asterisk. (e) Onset potential (defined as the potential to achieve a current of 0.1 mA cm^-2^) as a function of the position of the SWV peak. Single-peak molecules are shown with triangular symbols, while dual-peak molecules are shown with circles. The dashed line is added for visual aid and represents correspondance of the ORR onset with the SWV peak. (f) Square wave voltammogram of 8EtFeP in nitrogen-saturated (full line) and oxygen-saturated (dashed line) 0.1 M KOH. Data were collected in static conditions, using a cathodic scan, composed of a potential step of 4 mV, modulation amplitude of 20 mV, and a frequency of 2 Hz, resulting in a scan rate of 8 mVs^–1^.

To gain a better understanding of the nature of the cyclic voltammetry peaks and their relationship with the ORR activity, we selected a library of commercially available Fe macrocycles. The investigated macrocycles are depicted in Figure 1a.

Rather than using cyclic voltammetry (CV), we utilized square-wave voltammetry (SWV) to identify the position of the redox peaks. As shown in Figure 1b, SWV consists of a symmetrical square wave, superimposed on a base staircase potential. The current is sampled at the end of the forward step and at the end of the reverse step, and the difference between the two is plotted as a function of the applied potential. By performing this step process, it is possible to obtain high rejection of the capacitive current and better sensitivity to Faradaic processes compared to cyclic voltammetry. As a result, it is possible to observe redox peaks that would otherwise be buried in the capacitance and invisible in the cyclic voltammograms. An example of the higher sensitivity of SWV has been observed on a pyrolyzed catalyst, for which SWV uncovered the existence of Faradaic peaks invisible in the cyclic voltammogram (Figure S2).

A frequency of 2 Hz was found to offer a good balance between peak resolution, time, and signal-to-noise ratio and was used for all measurements of the Fe-macrocycles. The results of the cathodic scans in N_2_-saturated 0.1 M KOH are shown in Figure 1d, where the peak position has been highlighted by an asterisk (where available, the number and position of the peaks are in accordance with literature data, as shown in Figure S1g). Figure 1c shows the oxygen reduction activity of the same macromolecules in an O_2_-saturated 0.1 M KOH. From these results, it can be seen that, despite having the same FeN_4_ active site and loading, the selected macrocycles offer a broad range of ORR activity and SWV peak positions and sizes. Two more interesting observations can be made:

(1) Some molecules, i.e., FePC, 4COOHFePC, and 16ClFePC, exhibit two peaks in the potential range scanned, while the remaining ones (4PhFeP, 8EtFeP, 4NH_2_FePC) only present one peak. In this work, these two groups will be referred to as “dual-peak” and “single-peak”, respectively. Interestingly, the affiliation of macrocycles to either group is not controlled by the nitrogen ligand, as single-peak molecules feature both porphyrins and phthalocyanines. To confirm that the second peak isn’t outside of the potential window scanned, we repeated the scans extending the maximum potential until oxygen evolution would start or the electrode would visibly degrade, and no peak was observed (Figure S3).
(2) For the dual-peak molecules, the potential for a 0.1 mAcm^–2^ ORR current follows closely the position of the SWV peak, while the same is not true for single-peak molecules (Figure 1e). For example, for the case of 8EtFeP, shown in Figure 1f, in the absence of oxygen, a SWV peak can be observed at 0.3 V vs RHE, while an oxygen reduction current can be observed at a potential more than 500 mV more positive. If the SWV peak was due to *OH desorption, as previously suggested, it remains to be explained how oxygen reduction can take place at such high potentials, where the Fe active sites should be poisoned with strongly adsorbed *OH.

In the following sections, we will try to elucidate the nature of the redox peaks and explain the high activity of single-peak molecules.

All the cyclic voltammograms and square-wave voltammograms reported in Figure 1 have been reported using the IUPAC convention; the voltammogram shown is the second one, starting from 1 V vs RHE and scanning cathodically first. The working electrode is a 5 mm glassy carbon RDE, on which the specified catalyst has been deposited; the counter electrode is a graphite rod, and the reference electrode is Hg/HgO. However, results are plotted vs RHE without iR compensation (calibration details are available in the Materials and Methods). All of the measurements were collected at room temperature.

### The pH Effect: Distinguishing between Charge Transfer and Proton-Coupled Electron Transfer

**Figure 2:**
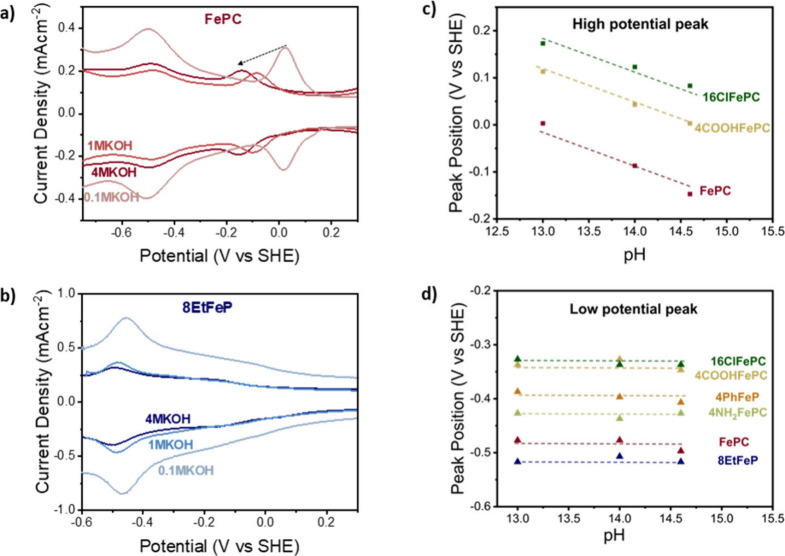
(a) Square wave voltammogram of FePC at different pH values: from light to dark red, data have been recorded in 0.1 M KOH, 1 M KOH, and 4 M KOH. As can be observed, the position of low potential peak remains unvaried with increasing pH, while the high potential peak shifts to lower potential. (b) Square wave voltammogram of 8EtFeP at different pH values: from light to dark blue, data have been recorded in 0.1 M KOH, 1 M KOH, and 4 M KOH. As can be observed, the only peak present does not move with pH. (c) Position of the low potential peak as a function of pH for the selected iron macrocycles. (d) Position of the high potential peak, as a function of pH, for the selected iron macrocycles All the square-wave voltammograms in this figure have been reported using the IUPAC convention, starting from 0.3 V vs SHE and scanning cathodically first. The working electrode is a 5 mm glassy carbon RDE, on which the specified catalyst has been deposited; the counter electrode is a graphite rod, and the reference electrode is Hg/HgO; however, results are plotted vs RHE without iR compensation (calibration details are available in the Materials and Methods). All the measurements were collected at room temperature.

A method to distinguish between Faradaic peaks resulting from charge transfer (e.g., Fe^+^ → Fe^2+^ + e^–^) and those involving a proton coupled-electron transfer (e.g., Fe^2+^ + H_2_O → Fe–OH + (H^+^ + e^–^)) consists of studying the SWV peak positions as a function of pH. To avoid introducing different cations whose effect cannot be entirely predicted,^51−55^ we varied the pH from 13 to 14.6 by changing the KOH concentration in the electrolyte and recorded the square wave voltammograms in N_2_-saturated electrolytes. Figure 2a,b shows the SWVs on the SHE scale with different KOH concentrations for FePC and 8EtFeP, respectively. As shown in Figure 2 for both FePC and 8EtFeP, the low potential peak position is unaffected by the change in pH, while in Figure 2a, showing FePC, the high potential peak moves to lower potentials as the pH increases. Figure 2c,d displays two Pourbaix diagrams (V vs SHE vs pH) of the positions of the high potential and low potential peaks, respectively, for all the selected molecules. As shown in Figure 2c, the high potential peak of the macrocycles showing 2 peaks depends on pH with a slope within experimental error to the expected 59 mV per pH unit, indicating that this peak is related to a proton-electron transfer. On the other hand, the low potential peak is related only to an electron transfer, as shown by its independence in pH (Figure 2d).

**Figure 3:**
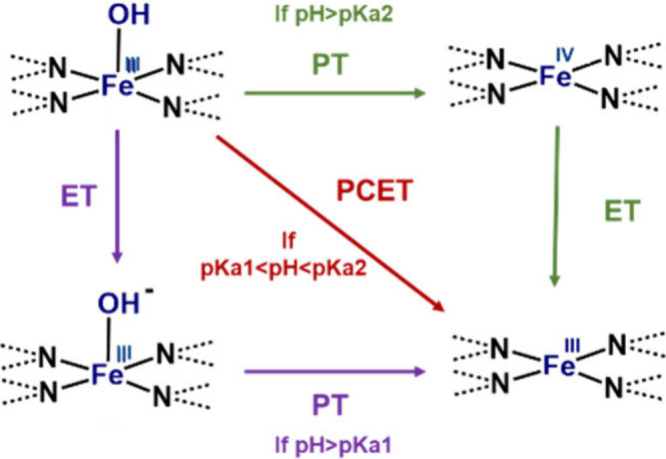
Schematic showing *OH desorption from a FeN_4_ active site in the case of a proton-coupled electron transfer (red arrow), proton transfer followed by an electron transfer (green arrow), and electron transfer followed by a proton transfer (purple arrow).

For the macrocycles only showing one peak (4PhFeP, 4NH_2_FePC, and 8EtFeP), this peak position is still pH independent, despite it having been previously attributed to *OH desorption.^45^ The pH independency of single-peak molecules suggests either that this is a simple electron transfer or that it is *OH desorption but proton and electron transfer are decoupled (Figure 3). This would be the case if the pH range tested was below the pKa of *OH desorption after an electron transfer to the iron center (pKa1 in Figure 3), meaning that the potential-determining step would be the initial electron transfer, which is pH-insensitive, followed by a thermodynamically driven *OH desorption. In theory, this mechanism could be confirmed by extending the pH range below the pKa. An extension of this study to a wider pH range could shine more light into the nature of the volammetric peaks and their effect on the ORR activity. However, this would require a careful consideration of possible specific adsorption effects from the salts or buffers in use. Moreover, it should also be considered that pH changes can alter the structure of functional groups and influence the ORR activity.

On a separate note, we would also like to point out that the magnitude of the voltammetric peaks often appears dependent on the electrolyte. Particularly, for the case of the low potential peak, which is attributed to a simple electron transfer, the size of the peak should be solely a function of the number of iron sites. As such, the peak size is also expected to be similar for all of the macromolecules considered for the same iron loading, but it instead appears to vary significantly. This phenomenon has been observed before without a universally accepted explanation^56,57^ but could be attributed to the difference in the relative magnitude of the capacitive current or in the accessibility of the iron sites. Finally, for this specific case, it should also be noticed that these measurements were performed in a large potential range, going above 1 V vs RHE, where carbon corrosion can take place at a rate that could be dependent on the electrolyte composition, potentially explaining the differences observed.^58,59^

### Confirming Iron Is the Active Site

**Figure 4:**
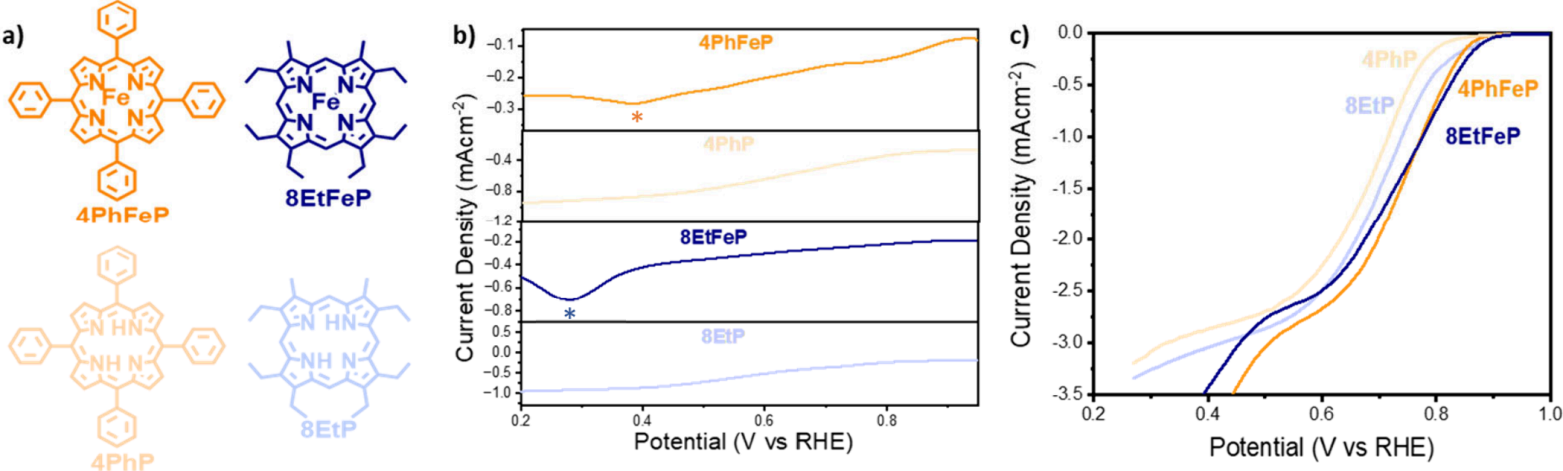
(a) Structure and abbreviation of the macromolecules 8EtFeP (blue), 8EtP (light blue), 4PhFeP (orange), and 4PhP (light orange). (b) Square wave voltammogram of 8EtFeP (blue), 8EtP (light blue), 4PhFeP (orange), and 4PhP (light orange) in N_2_-saturated 0.1 M KOH. Data were collected in static conditions using a potential step of 4 mV, modulation amplitude of 20 mV, and a frequency of 2 Hz, resulting in a scan rate of 8 mVs^–1^. The position of the identified peaks is shown with an asterisk. (c) Linear sweep voltammogram of 8EtFeP (blue), 8EtP (light blue), 4PhFeP (orange), and 4PhP (light orange) obtained in O_2_-saturated electrolyte with a scan rate of 10 mVs^–1^ at a rotational speed of 1600 rpm. All the cyclic voltammograms and square-wave voltammograms reported in this figure have been reported using the IUPAC convention; the voltammogram shown is the second one, starting from 1 V vs RHE and scanning cathodically first. The LSVs are scanned cathodically starting from 1 V vs RHE. The working electrode is a 5 mm glassy carbon RDE on which the specified catalyst has been deposited; the counter electrode is a graphite rod, and the reference electrode is Hg/HgO; however, results are plotted vs RHE without iR compensation (calibration details are available in the Materials and Methods). All the measurements were collected at room temperature.

The ORR activity of single-peak molecules at a potential where Fe should be blocked with adsorbed *OH could be easily explained if Fe was not the active site. After all, we are testing the ORR in alkaline conditions, where N/C catalysts have been reported to offer decent activity. To assess this possibility, we tested the electrochemical performance of the same porphyrin macrocycles in the absence of the metal center. On the metal-free macrocycles, the SWV peak in the N_2_ saturated electrolyte (at 0.4 and 0.3 V vs RHE, for 4PhFeP and 8EtFeP, respectively) disappears, confirming that the peak originates from the Fe center (Figure 4b). Figure 4c shows the oxygen reduction activities of these macrocycles. For both 4PhFeP and 8EtFeP, the ORR activity is diminished in the absence of iron, suggesting that the metal center is the active site. It should be noted that the metal-free macrocycles still offer moderate ORR performance, in accordance to previous reports of N–C materials as ORR catalysts in alkaline media.^60^ Finally, Zúñiga and co-workers reported a similar ORR activity decrease after poisoning the metal center of the same molecules (4PhFeP and 8EtFeP) with KCN in alkaline conditions,^50^ and the same behavior was observed by Yang and coworkers when poisoning a pyrolized FeN_4_ catalyst with KSCN,^61^ among others.^62^ These observations, in addition to the experimental data collected in this work, suggest that iron is the active site for the case of both porphyrins, while the state of the metal center during the reaction (i.e., the oxidation state and presence of adsorbed species) remains to be determined.

### Tafel Analysis

**Figure 5:**
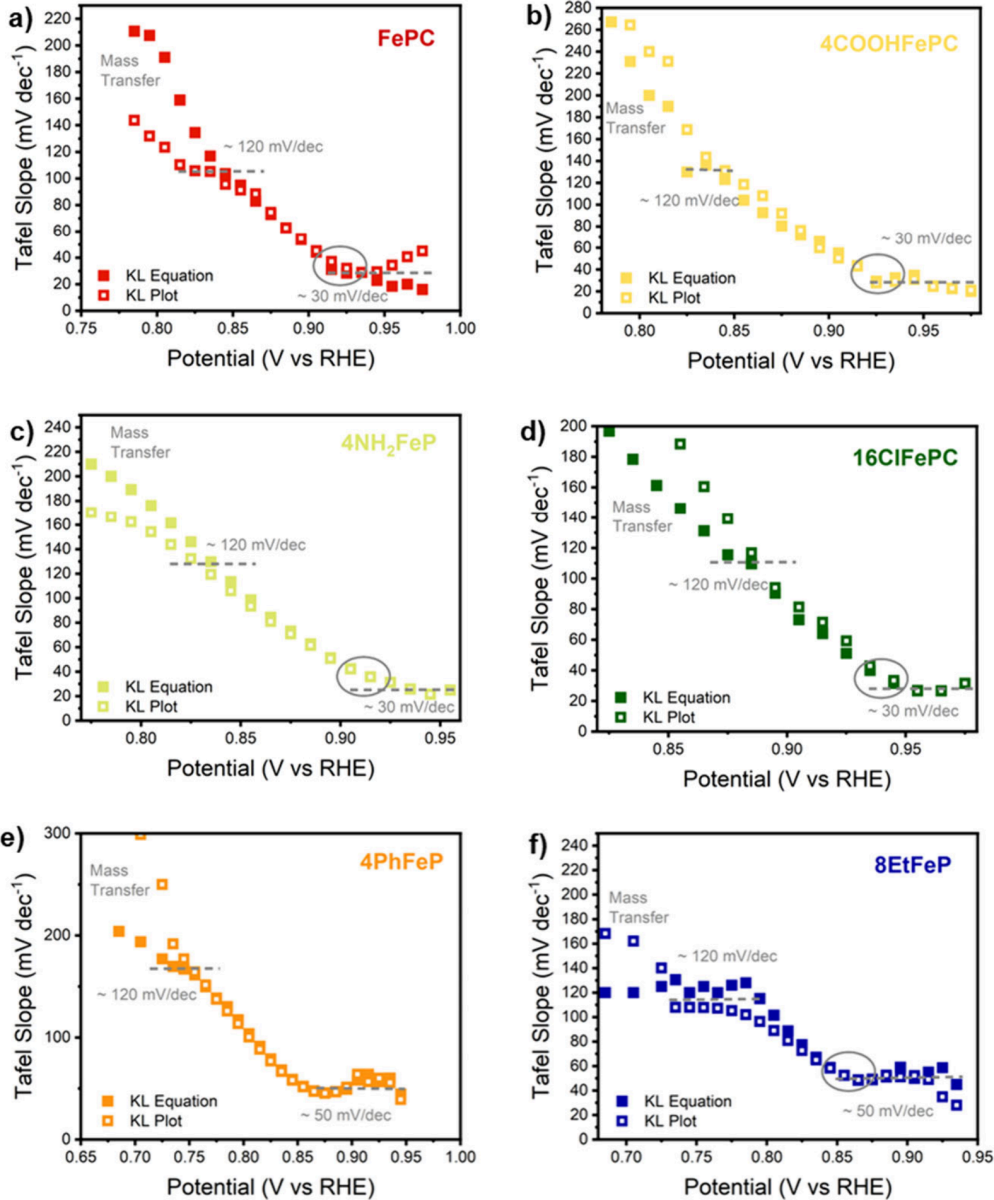
Tafel slope of the selected macrocycles as a function of the applied potential. The Tafel slope was determined from the derivate of the Tafel plot, i.e., the plot of the applied potential as a function of the logarithm of the kinetic current. The kinetic current was determined using either the Koutecky-Levich equation (full symbols) or the Koutecky-Levich plot (empty symbols), as detailed in the Supporting Information.

The Tafel analysis can be used to identify changes in the potential-determining step and to assist in the identification of these steps. This analysis starts by plotting the applied potential as a function of the logarithm of the kinetic current density (Figure S5), also known as Tafel plots. The slope of the resulting curve, referred to as the Tafel slope, is a measure of the current sensitivity to the applied potential and is what is used to determine the nature of the potential-determining step. Generally, Tafel slopes are determined by fitting multiple linear regions of the Tafel plot. However, Tafel analysis has multiple limitations. On one hand, various linear regions can be identified, making the fittings subjective and resulting in several Tafel slopes having been reported for the same catalyst. On the other hand, a meaningful Tafel slope relies on the accurate extraction of the kinetic current, which is not always straightforward.^63^ To be able to reliably and objectively distinguish linear fundamental regions of the Tafel plot from non-kinetic processes, we followed the approach proposed by Koper and co-workers.^63^ This consists of plotting Tafel slopes over small intervals, as a function of the applied potential, removing the need for an a priori determination of linear regions of the plot. Furthermore, to reliably extract the kinetic current, both the Koutecky-Levich equation (full symbols) and the Koutecky-Levich plot (empty symbols) were used and compared (details in Section S2). Figure 5 shows the results for all of the molecules considered in this work. As it can be observed, all the macrocycles display an initially linear region (around 30 to 50 mV/dec), followed by a gradual increase of the Tafel slope. It can also be noticed that, at low overpotentials, the Tafel slope is independent of the technique used to extract the kinetic current. On the contrary, when the Tafel slope reaches 120 mV/dec, the results obtained with the KL plot and the KL equation start to deviate, indicating the presence of non-kinetic processes, likely due to mass transport limitations. The fact that the deviation always occurs above 120 mV/dec suggests that this is the intrinsic value of the Tafel slope at high overpotential, even though the presence of transport limitations prevents us from drawing definitive conclusions in the high-current region. What we can conclude from the results is that all the molecules start from a low Tafel slope of 30 to 50 mV/dec, which starts to gradually increase at potential lower than 0.85–0.95 V depending on the molecule, in line with previous reports.^64−66^

Changes in the Tafel slope with the applied potential can be caused by changes in the rate-determining step with potential, superposition of different sites contributing to the overall activity, or changes in coverage of the rate-limiting species. Given the observed increase in Tafel slope with overpotential, we conclude that changes in coverage are the most likely explanation (more details in Section S2).

Following previous reports^30,66,67^ and the insights collected in this work, we hypothesize the existence of an equilibrium step, determining the gradual decrease of FeOH species (eq 1), followed by a slow *OOH formation or by fast O_2_ adsorption and a slow electron transfer (eq 2)
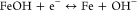
1
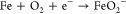
2

Assuming these steps, we derived an expression for the current as a function of the applied potential, as shown in eq 3 (details can be found in Section S2).
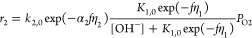
3where η_1_ and η_2_ are the overpotentials for steps 1 and 2, respectively, *f* = *F*/*RT*, where *F* is the Faraday constant, *R* is the gas constant, and *T* is the temperature. *K*_1,0_ is the equilibrium constant for step 1; α is the symmetry factor, and *P*_O2_ is the oxygen partial pressure.

From this expression, one can predict the behavior at the extreme cases of high and low overpotentials. At high overpotentials, the coverage of free Fe sites tends to 1, and the expression simplifies to *r*_2_ ∼ *k*_2,0_ exp(−α_2_*f*η_2_)*P*_O2_, predicting a Tafel slope of 120 mV dec^–1^. On the contrary, at low overpotentials, the reaction rate is controlled by the concentration of free Fe sites, leading to an expected Tafel slope of 40 mV dec^–1^.

Figure S5 shows the simulated change of Tafel slope as a function of overpotential. The Tafel slope can be seen to gradually increase from 40 to 120 mV dec^–1^ over a potential window of about 100 mV in concomitance with the formal potential for *OH desorption. This is in line with our experimental observations^44^ and would mean that the deviation from the initial Tafel slope of 40 mV/dec can be used to experimentally observe *OH coverage and measure the *OH binding energy. For the case of dual-peak molecules, the change in Tafel slope does indeed accurately follow the high potential redox peak in nitrogen (also an experimental measure of the *OH binding energy). For single-peak molecules, the same behavior is observed, suggesting a similar mechanism involving a change in coverage of a species involved in the rate-determining step, possibly adsorbed *OH. Interestingly, this change happens in the range of 0.8 to 0.9 eV, where one would expect *OH desorption to happen given the ORR performance of these molecules.

### Peroxide Selectivity Is Controlled by the Presence of Adsorbed Water

We then used a rotating-ring disc electrode to elucidate the selectivity of oxygen reduction on the selected macromolecules. Once the *OOH intermediate is formed, the reaction can follow two different pathways, one involving the desorption of HOO^–^ and one implicating the dissociation of the O–O bond, leading to the 4e^–^ reduction to water (eq 4).
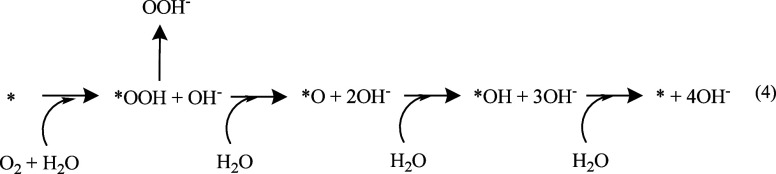
4

**Figure 6:**
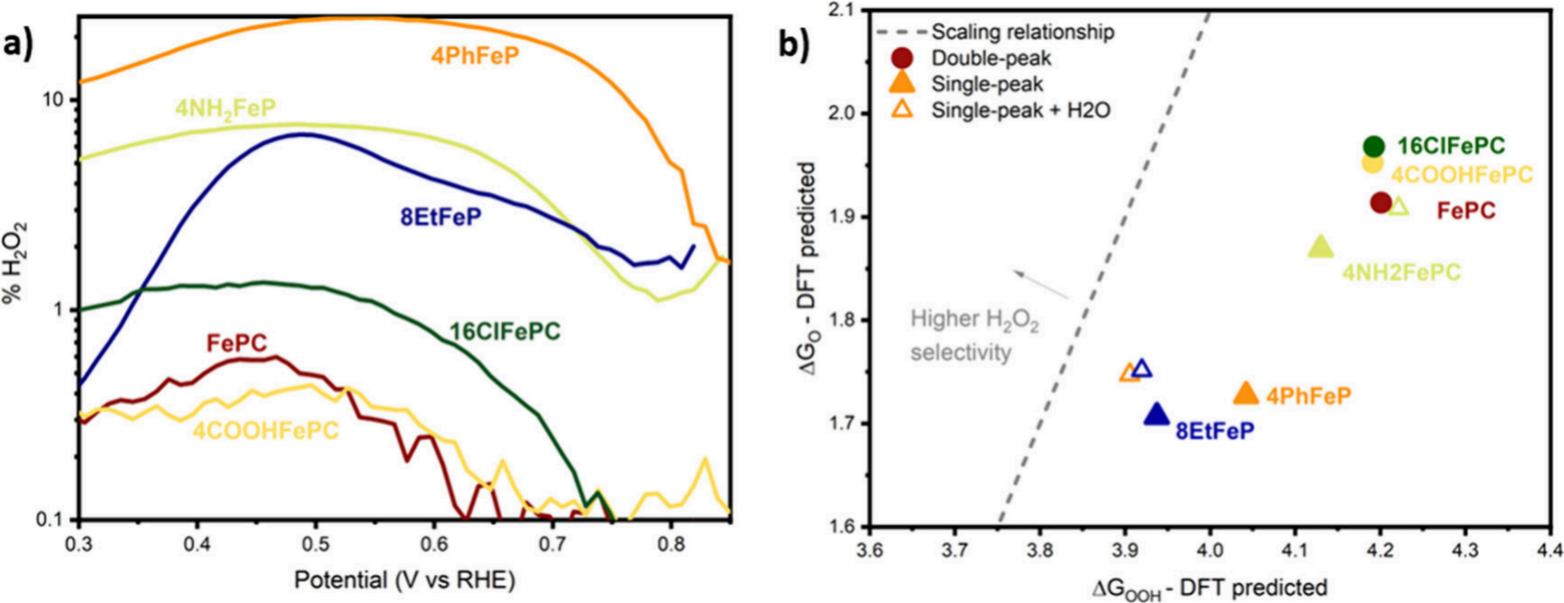
(a) Peroxide selectivity for all the macrocycles considered in this manuscript. Data were collected using a RRDE setup, at 1600 rpm, in O_2_-saturated 0.1 M KOH. Data were collected starting from 1 V vs RHE and scanning cathodically. The working electrode is a 5 mm glassy carbon RRDE with a platinum ring on which the specified catalyst has been deposited, the counter electrode is a graphite rod, and the reference electrode is Hg/HgO; however, results are plotted vs RHE without iR compensation (calibration details are available in the Materials and Methods). All the measurements were collected at room temperature. (b) *O binding energy, as a function of *OOH binding energies, as predicted by DFT calculations. Circles represent double-peak molecules, while triangles are used for single-peak molecules. For single-peak molecules, the simulation results with back-side adsorbed water are also shown as empty triangles. The dashed line shows the binding energy predicted by the scaling relationship Δ*G*_O_ = 2Δ*G*_OOH_ – 5.9, which was derived for metal porphyrins and functionalized graphitic materials.^38^

Earlier in this article, we discussed how the scaling relationship among the binding energies of oxygenated intermediates leads to a Sabatier volcano, where the binding energy of a single intermediate is sufficient to predict the theoretical minimum overpotential.^68^ These electronic effects control completely the catalytic activity and partially the selectivity. For the case of water production, *OH desorption limits the activity on the strong-binding (left) side of the volcano, while O_2_ reduction to *OOH is the limiting factor on the opposite side. On the other hand, peroxide production is limited by *OOH desorption on the left side of the volcano and again O_2_ reduction to *OOH on the right side. Since the limiting potential for *OH desorption is always more positive than *OOH, water production is always favored over peroxide production on the left side of the volcano. Since all of the molecules tested are expected to be on the left side of the Sabatier volcano (as also confirmed by DFT simulations, discussed below), we would a priori expect them to exhibit low peroxide selectivity. Figure 6a shows the peroxide selectivity as a function of the potential. While, as expected, all the double-peak molecules offer low peroxide selectivity (below 0.5% over the whole potential range), single-peak molecules are much more efficient at producing hydrogen peroxide (with a selectivity of up to 25%). This difference in selectivity implies that the electronic effects that lead to the volcano cannot fully capture the performance under ORR conditions.

An earlier study (co-authored by one of us) showed that trends in selectivity could be described by the binding energy of the *O intermediate with respect to *OOH: binding O* strongly relative to *OOH is necessary in order to dissociate *OOH.^69^ To assess whether this effect could be responsible for the unexpected peroxide selectivity trends, we determined the *O and *OOH binding energies using DFT (discussed in more detail in the next section), and the results are plotted in Figure 6b. All the analyzed Fe-macrocycles show a stronger *O bond than expected from the scaling relationship (Δ*G*_O_ = 2Δ*G*_OOH_ – 5.9,^38^ dashed line in Figure 6b) and lie on a similar line. Therefore, we additionally considered the possibility of the single-peak macrocycles having an axial ligand such as water. As can be noticed, the presence of backside adsorbed water has a small effect on the *O bond, while it strongly affects the *OOH bond, strengthening it for some macrocycles and weakening it for others. This ligand would cause 4PhFeP and 8EtFeP to lie closer to the predicted line, meaning that they have a weaker *O bond for the same *OOH bond compared to that of the phthalocyanine. This means that the presence of an oxygenated ligand could explain their high peroxide selectivity. 4NH_2_FePC is the only exception, as it lies right on the graph but displays good peroxide selectivity.

### Single-Peak Macrocycles Show No Spectral Change in *Operando* XAS

**Figure 7:**
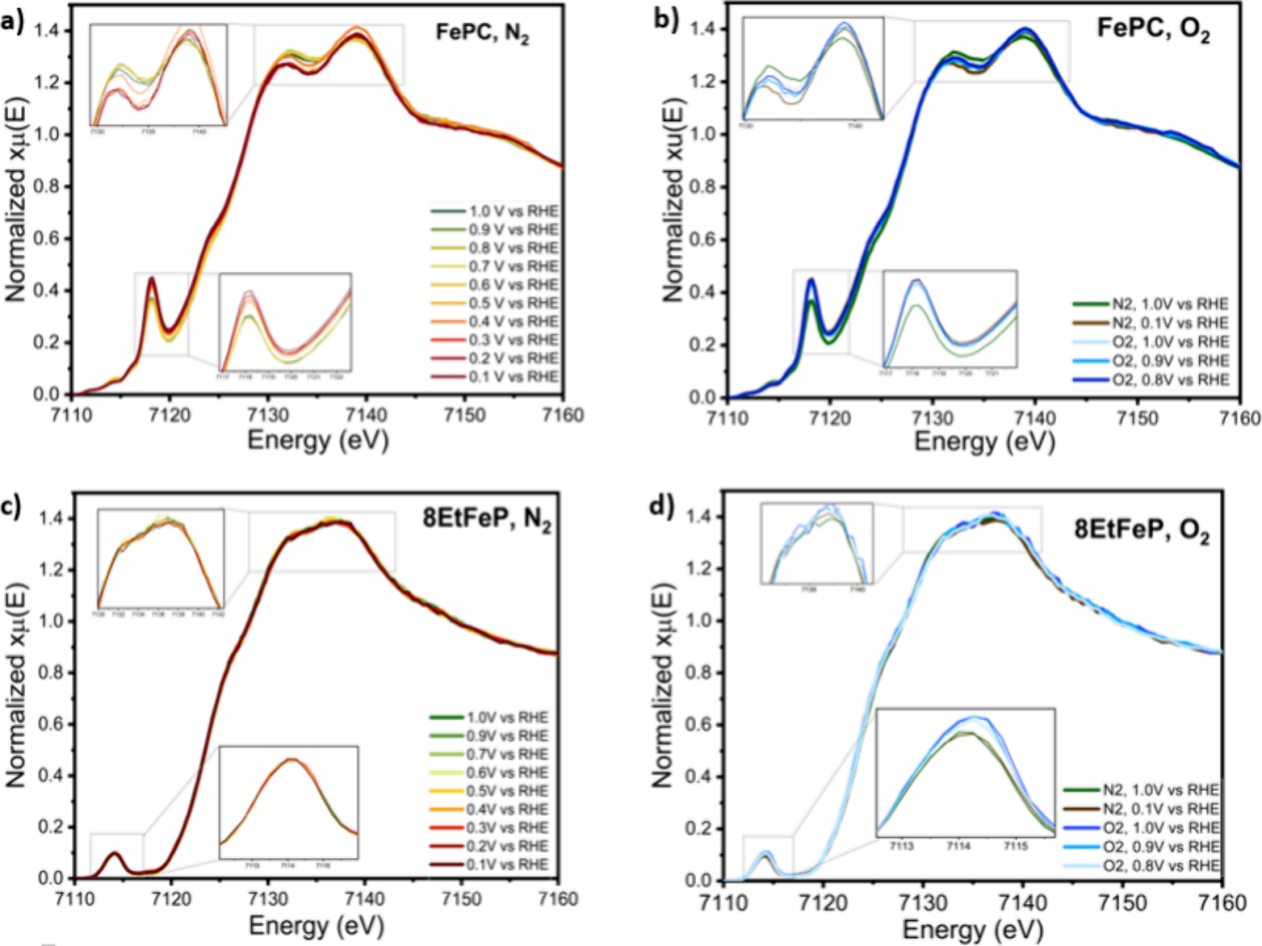
Operando Fe Kα HERFD XANES spectra, obtained with the custom-made GDE cell described in the Supporting Information. (a) FePC with flowing nitrogen, going from green to red, the plots represent the spectra under an increasingly reducing potential, from 1 to 0.1 V vs RHE. The insets show a zoom-in of the peak and pre-peak. (b) XAS spectra of FePC under an oxygen flow. From light blue to dark blue, the spectra are obtained under oxygen at a potential of 1, 0.9, and 0.9 V vs RHE, while green and brown show spectra in nitrogen at 1 and 0.1 V vs RHE for comparison. (c) 8EtFeP with flowing nitrogen; going from green to red, the plots represent the spectra under an increasingly reducing potential from 1 to 0.1 V vs RHE. The insets show a zoom-in of the peak and pre-peak. (d) XAS spectra of 8EtFeP under an oxygen flow. From light blue to dark blue, the spectra are obtained under oxygen at a potential of 1, 0.9, and 0.9 V vs RHE, while green and brown show spectra in nitrogen at 1 and 0.1 V vs RHE for comparison.

To gain a better understanding of the change in the structure of the iron macrocycles with potential, we observed the HERFD XANES (high-energy-resolution fluorescence-detected X-ray absorption near edge structure) spectra of selected molecules upon *operando* modification of the applied potential. To do so, a custom-built, gas diffusion electrode cell was employed, and selected molecules (FePC, 4PhFeP, 8EtFeP, and 4NH_2_FePC) were studied while flowing oxygen or nitrogen (experimental details can be found in Section S3). The XANES spectra collected at OCP are consistent with those previously reported.^70−72^ The spectra of all molecules did show a change with potential when in oxygen-saturated conditions, suggesting once again that iron is the active site and confirming the validity of the operando setup (Figure 7, Figure S9). FePC, which is a double peak molecule, showed a spectral change with the applied potential in the N_2_-saturated electrolyte. As shown in Figure 7a, this change happens around the high potential peak and is characterized by a shift of the white line to higher energy values, a decrease of the white line intensity, and a reduction of the intensity of the pre-edge peak located at 7118 eV. At the same time, all of the single-peak macromolecules do not display any potential-dependent spectral change in N_2_-saturated electrolyte, as shown in Figure 7c for the case of 8EtFeP.

Mukerjee and co-workers performed similar *in situ* XAS studies on iron macrocycles, after subjecting them to heat treatment at different temperatures.^72−74^ In multiple articles, they reported a change in the white line intensity in concomitance with the high-potential redox peak, which was attributed to a change in the oxidation state. In accordance to our observations, they reported this change for FePC pyrolyzed at 300 and 800 °C in both acidic and alkaline electrolytes.^72^ On the contrary, they also reported a similar spectral change for 4PhFeP heat-treated at the same temperature in 0.1 M HClO_4_,^73,74^ which was not observed here. This suggests that either the heat treatment or the electrolyte influences the reaction mechanism for these molecules, which warrants further studies.

Returning to the case of unpyrolyzed macrocycles in alkaline conditions, studied here, we simulated the XAS spectra of the macrocycles in the presence of different adsorbed species to understand the difference among these molecules and the reason behind the lack of a spectral change (Figure S10). Among the simulated spectra, those of OH–Fe–H_2_O offer the best match for 4PhFeP and 8EtFeP, while the water-free spectra (Fe^2+^ and Fe^3+^ especially) more closely follow the experimental data obtained for FePC. In particular, the simulations suggest that all molecules should present a pre-edge peak at 7118 eV, which disappears in the presence of adsorbed water. This pre-edge peak is indeed observed at every potential for FePC, while it is absent for 4PhFeP and 8EtFeP, again suggesting the presence of an oxygenated adsorbate for the last two molecules. For the case of 4NH_2_FePC, the pre-edge peak at 7118 eV is absent, but the simulated spectra do not accurately match the experiments; thus, no definitive conclusion can be established.

This analysis highlights a further difference between single-peak and dual-peak molecules and provides an indication that an oxygenated adsorbate could be present on single-peak molecules. The implication of this will be further elaborated on in the discussion. In brief, this suggests that the presence of an oxygenated intermediate in single-peak molecules could accommodate the spin of Fe, allowing the *OH desorption to happen in the absence of an electron transfer. This could explain the presence of a single redox peak and also the impressive difference in the ORR activity between single- and dual-peak molecules.

### DFT Simulations

Finally, we used DFT modeling to understand the difference in activity and selectivity between single- and dual-peak macromolecules. The well-defined macrocycles are a particularly good system to investigate metal nitrogen carbon catalysts, as the exact structural motif can be used directly in density functional theory (DFT) simulations to gain a one-to-one description of experiments and simulation. Here, we applied DFT simulations to predict the activity of the macrocycles by determining the binding energies of different ORR intermediates, *O, *OH, *OOH, and, when possible, comparing them with the high potential peak from the SWV curves.

The simulations on the pristine iron macrocycle (i.e., without intermediate) show two stable magnetic structures of each iron macrocycle after spin-relaxed optimizations. One has a magnetic moment of zero, which resembles Fe^2+^. The other one is near two and can be inferred as Fe^3+^ by DFT and experimental data (Section S4). Interestingly, even though original species have different spins, only one spin state is possible once intermediates are added. This is backed by the convergence of different initial states. The calculated binding energies of the ORR intermediates are summarized in Table S1. It should also be noticed that 8EtFeP has isomers marked with AABB and ABAB indicating the arrangements of the ethyl group. The results of both are included in the Supporting Information, but for simplicity, both data points are referred to as 8EtFeP in the figures.

As the pristine macrocycles display two spin states, while intermediates only have one, *OH desorption can be calculated for both spin states accordingly:
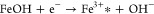
5
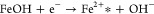
6

We do not know which state (Fe^2+^ or Fe^3+^) the macrocycles are in when the *OH desorption involving the proton-electron transfer takes place, but the further reduction of Fe^2+^ would be to Fe^+^ and the further reduction of Fe^3+^ would be to Fe^2+^.

**Figure 8:**
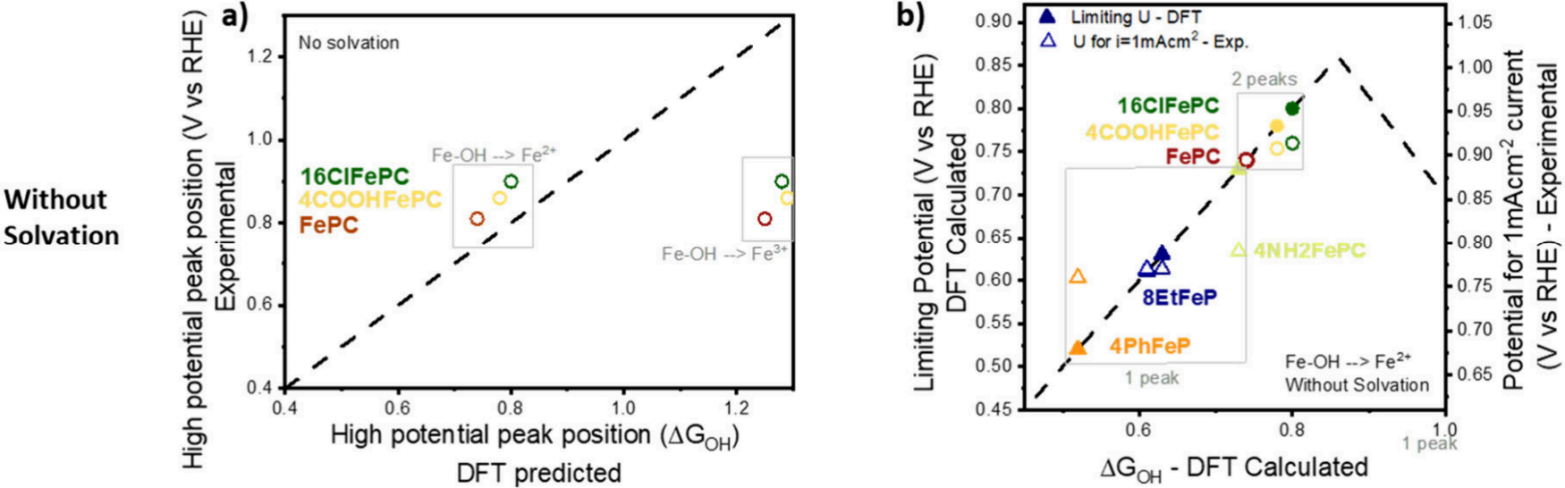
(a) Comparison of high-potential peak positions, obtained experimentally and using DFT calculations, omitting any additional solvation effect. The experimental peak position is only shown for the molecules displaying two peaks and was determined as the average of the SWV high peak position in the cathodic and anodic scan. (b) Volcano plot showing on the left *y*-axis the DFT-limiting potential (full symbols) for oxygen reduction and on the right *y*-axis the experimentally determined potential necessary to reach a current density of 1 mAcm^–2^. Both data sets are plotted as a function of the DFT-predicted *OH binding energy. The DFT calculations are obtained assuming that OH desorption happens with a change in the Fe oxidation state: Fe–OH to Fe^2+^ and omitting solvation corrections. The experimental data are scaled to fit the prediction for FePC.

Figure 8 shows the simulated *OH binding energy in direct comparison with the experiments. To take into account the effect of solvation, we first applied a 0.3 eV correction (value normally applied for platinum metal surfaces^75^), but this value failed to represent the experimental results (Figure S12c,d). Finally, we considered the case of no correction and 0.45 eV correction, which allows one to correctly identify the high peak position of FePC, for the case of Fe–OH to Fe^2+^ and Fe–OH to Fe^3+^, respectively. Notably, DFT simulations are robust when observing relative energetics for such systems as macrocycles, whereas the solvation and absolute energetics come with much higher uncertainty. In the case here, with close energetics, we therefore focus on comparing relative energetics to FePC from both the computational and experimental sides.

Figure 8a compares the experimental high peak position of 16ClFePC, 4COOHFePC, and FePC (the dual-peak molecules) with calculated *OH binding energy from Fe–OH to Fe^2+^ (*m* = 0) and Fe^3+^ (*m* = 2) in the absence of solvation corrections. The three experimental points fall close to the diagonal in a trend wise fashion with respect to the simulated *OH binding energy from Fe–OH to Fe^2+^ (*m* = 0), while the Fe–OH to Fe^3+^ (*m* = 2) calculated *OH has too high energetics. Matching three experimental data points with three simulated data points may limit robust conclusions due to limited data, so the comparison has been extended in the Supporting Information (Figure S11) to all the macromolecules, using the prediction of the *OH desorption potential obtained from the Tafel plot analysis presented above. A similar analysis has been repeated (Figure S10) using an energy correction of −0.45 eV, depicting that in this case the Fe–OH to Fe^3+^ (*m* = 2) calculated *OH could resemble the proton-electron-dependent peak. What it ideally shows is that the actual *OH desorption/adsorption prediction is independent of oxidation state with a given absolute DFT correction.

Figure 8b shows a volcano plot obtained using the calculated *OH binding energy from Fe–OH to Fe^2+^ (*m* = 0) (the equivalent for different solvation and oxidation states can be found in Figure S12). The DFT-predicted overpotential is on the left *y*-axis (full symbols) and is shown as a function of the DFT-predicted *OH binding energy. The experimental ORR activity is overlaid on the right *y*-axis (empty symbols) as a function of the DFT-predicted *OH binding energy by scaling the potential at 1 mAcm^–2^ current for FePC. This allows for a robust comparison between relative calculated DFT energetics and experimental performance.

Two observations can be made from this analysis in both figures. First of all, the calculated *OH from DFT allows us to group the two types of catalyst experimentally; the ones with two peaks are active with optimal *OH binding energies for ORR, and the ones with one peak have weaker *OH binding energy and are also much less active in the experiments. Secondly, the match between the *OH prediction and the overlay of the experimental performance shows that the dual-peak catalysts are predicted relatively well on the volcano, while the single-peak catalyst is poorly described in the volcano framework. This observation suggests that the single-peak molecules could follow a different activity mechanism.

Since it is impossible to determine the correct solvation value a priori, our analysis tells us that both mechanisms (5) and (6) are plausible. In situ XAS studies in this (Figure 7a) and previous works^10,39,46^ have reported a shift in the white line position at the high peak potential. This phenomenon has usually been attributed to a change in oxidation state of iron but could also result from *OH desorption while maintaining the Fe oxidation state at 3+. To the best of our knowledge, no other experimental evidence has been previously reported for the Fe^3+^/Fe^2+^ transition happening in concomitance with the high potential peak.

## Discussion

To summarize the observations we have collected so far, single-peak and dual-peak molecules appear to form two families of macrocycles with very different oxygen reduction activity and selectivity. Simulations of the *in situ* XAS spectra suggest the presence of an oxygenated intermediate for the case of single-peak molecules, responsible for the suppression of the pre-edge at 7118 eV, which is instead present in dual peak molecules. This intermediate could be adsorbed water, as recently suggested by Chen et al.,^76^ or *OH, as will be discussed below. The presence of such a back-side adsorbent could additionally explain the difference in peroxide selectivity between single-peak and dual-peak molecules. In particular, we attribute it to the differences between the FeN_4_ distortion angle in the presence and absence of a backside axial ligand,^76^ which, according to our DFT simulations, would unevenly affect the binding energy of different intermediates, weakening the *OOH bond more than the corresponding *O bond.

Another difference that was noticed among the macrocycles is that, while the XAS spectra of dual-peak molecules show potential-dependent changes in N_2_-saturated electrolyte, single-peak molecules do not. In addition, DFT simulations of the *OH desorption peak position and ORR activity are in good agreement with dual-peak molecules but not with single-peak ones, further suggesting that they might follow a different mechanism. Since the high-potential voltammetric peak has been previously attributed to *OH desorption, its position can be used as an experimental estimate of the *OH binding energy. When comparing this experimental value to the DFT-predicted *OH binding energy, we can once again observe a good agreement for dual-peak molecules but a very poor one for single peak molecules. This suggests that the redox peak observed in single-peak molecules might not be *OH desorption, as further supported by its pH independence. The last but possibly most important observation we collected for single-peak molecules is that an oxygen reduction current can be observed at potential 500 mV more positive than the redox peak, where the FeN4 centers should be poisoned by adsorbed *OH, and hence inactive.

**Figure 9:**
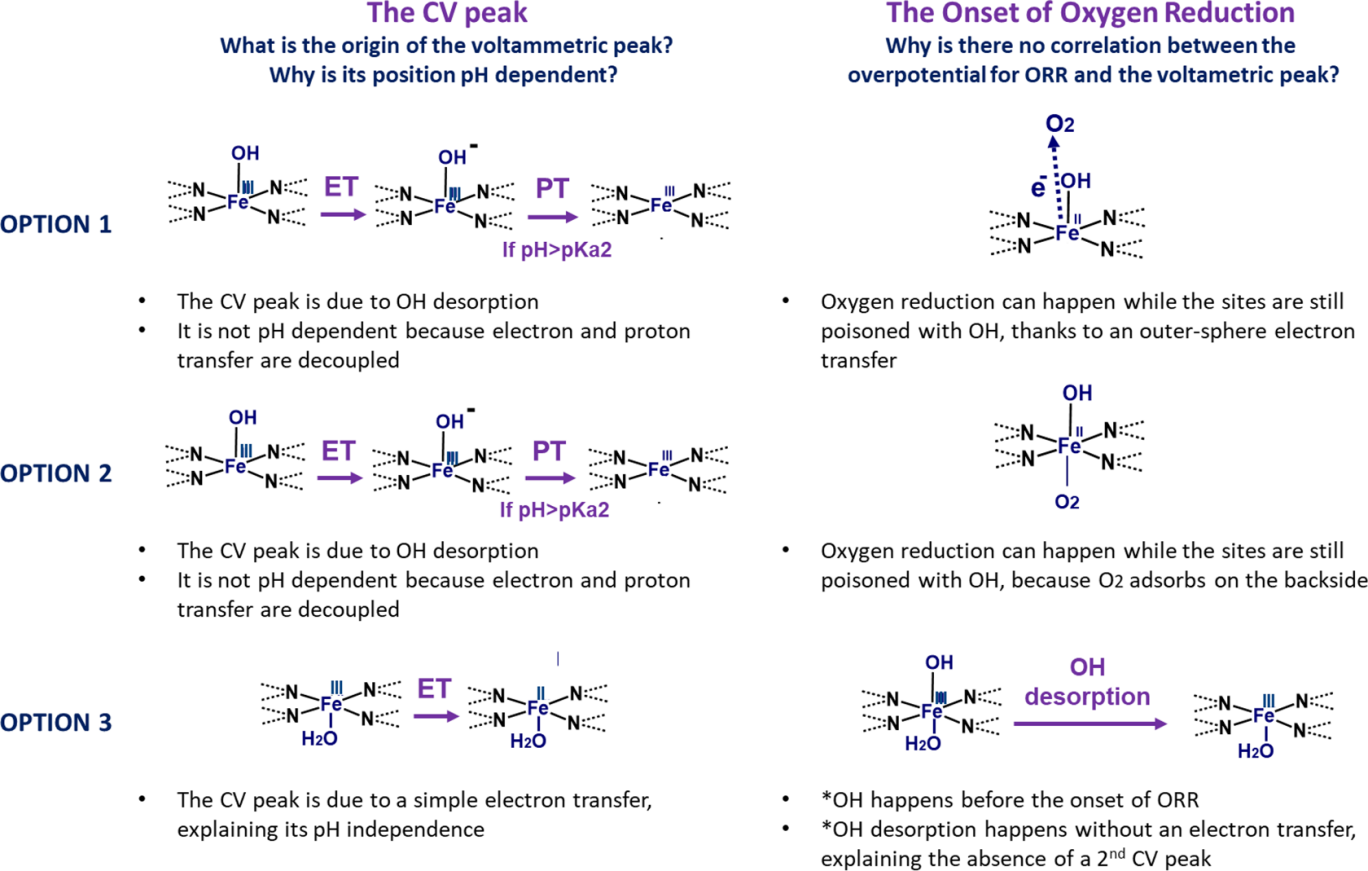
Three possible explanations identified to explain the discrepancies with single-peak macromolecules: the absence of a high potential voltammetric peak attributable to *OH desorption, the pH independence of the only voltammetric peak visible, and the observation of an ORR current at potentials 500 mV higher than the voltammetric peak, where the active site should be poisoned by *OH.

In light of all of the experimental results presented above, we propose three options for the ORR mechanisms on single-peak Fe macromolecules. These are summarized in Figure 9, where the nature of the redox peak and its pH dependence are explained on the left column, while the presence of oxygen reduction current above the potential of this peak is shown in the right column.

The first explanation involves a so-called outer-sphere electron transfer, which would allow the first electron transfer step to happen while *OH is still adsorbed on the Fe center (Figure 9 – option 1). In this mechanism, the electron is transferred to species near the outer Helmholtz plan, instead of being transferred directly to a strongly bound adsorbate. In the case of ORR, this mechanism would involve the reduction of O_2_ to O_2_^–^ through an outer-sphere electron transfer, followed by a proton and electron transfer to O_2_^–^ from the neighboring water molecules, forming adsorbed HOO^–^. This possibility has already been proposed for the case of platinum, gold, and Fe–N–C^77−79^ and would explain most of the observations, including the lack of potential-driven XAS changes and the higher peroxide selectivity. Nevertheless, a few remarks make this option unlikely in our opinion:

1. The outer-sphere electron transfer can only produce peroxide if *OH is desorbed. While the peroxide production experimentally determined is higher for single-peak molecules compared to dual-peak ones, it still is below 25% (Figure 7).
2. The first step of the outer-sphere electron transfer mechanism is an electron transfer to oxygen, to produce O_2_^–^, which has a very high energy barrier of 0.5 eV.^36^ Koslowski et al.^36^ have recently used DFT simulations to compare the energy requirement of the mechanisms involving outer-sphere and inner-sphere electron transfer on metal porphyrins. They concluded that, while the outer-sphere pathway is likely to happen on various metals, such as Co, for the case of Fe, the inner-sphere mechanism has a lower energy barrier.
3. If oxygen reduction followed an outer-sphere electron transfer, one would expect a constant Tafel slope, at least in the potential region above the low-potential voltammetric peak (0.3 to 0.4 V vs RHE). On the contrary, a clear change in Tafel slope is observed at high potential (0.8 to 0.9 V vs RHE – Figure 5b)

The second explanation for the detection of the ORR current above the *OH desorption potential is that oxygen reduction could take place at the back-side of a pentacoordinated FeN_4_–OH (Figure 9 – option 2). In this case, desorption of the second *OH ligand from HO–FeN_4_–OH would have to be thermodynamically favorable in the potential window scanned to explain the lack of an associated SWV peak. This mechanism has also been proposed before for the case of FeN/C and FeN_4_ macrocycles, mainly in alkaline conditions.^80−82^

While the possibility of a spectator backside *OH is, in our opinion, more likely than the outer-sphere electron transfer, it similarly fails to explain the change in Tafel slope with potential.

Finally, the third option involves *OH desorbing without electron transfer (Figure 9 – option 3). This would mean that ORR can take place on a free FeN_4_ site, and it would mean that the SWV peak would be caused by a simple electron transfer, with a change in iron oxidation state. As suggested by the XAS spectral simulations, it is possible that, during *OH desorption in the single peak molecules, an oxygenated ligand such as water could act to accommodate the spin of Fe instead of ordinary electron transfer. However, it still remains counterintuitive why *OH desorption is potential-dependent despite the absence of an electron transfer. This could be possible if potential-dependent changes in functional groups on the carbon support, or on the nitrogen sites,^79^ caused a gradual change in the electron density on the metal center, which would finally cause spontaneous *OH desorption. Another possibility is that *OH desorption could be caused by a local pH change induced by the formation of the electrical double layer.

This option would explain the absence of a *OH desorption peak in single-peak molecules and the pH independence of the observed redox peak, attributed to a simple electron transfer. In this case, the presence of adsorbed water would explain the observations regarding XAS and peroxide selectivity, and we conjecture that it would also be the determining factor to assign a macromolecule to the single-peak or dual-peak groups. Finally, compared to the other mechanisms, option 3 would also explain the change in the Tafel slope, which is assigned to the change in the rate-determining step from *OH desorption equilibrium to oxygen adsorption. If this was the case, the change in Tafel slope would be an experimental measure of *OH desorption and hence of its binding energy. This value would additionally be in good accordance with the DFT-predicted *OH binding energies (Figure S11).

## Conclusions

In this paper, we have used the well-defined FeN_4_ sites offered by Fe-macrocycles to study the oxygen reduction mechanism and observe the effect of ligands. Square-wave voltammetry was used to accurately identify the redox peaks present in nitrogen-saturated conditions; it revealed the existence of two families of Fe macrocycles: single-peak and dual-peak molecules, showing one or two voltammetric peaks, respectively. In addition to the voltammetry, other characteristics such as catalytic activity and selectivity distinguish these groups, which were summarized in Table 1. For example, single-peak molecules show poor oxygen reduction activity, high peroxide production, and no potential-dependent change in the XAS spectra in nitrogen-saturated electrolyte, while the opposite is true for dual-peak molecules.

**Table 1:** Summary of the Characteristics of the Fe Macrocycles Analyzed in This Manuscript^a^

Molecule	High Potential Peak	Change in XAS Spectra	Peroxide Selectivity	ORR Activity	Position on the Volcano
4NH2FePC	No	No	High	Low	Strong binding
4PhFeP	No	No	High	Low	Strong binding
8EtFeP	No	No	High	Low	Strong binding
4COOHFePC	Yes		Low	High	Strong binding
FePC	Yes	Yes	Low	High	Strong binding
16ClFePC	Yes		Low	High	Strong binding

a The change in XAS spectra refers to the observation of a spectral change upon *in situ* application of a potential from 1 V vs RHE to 0.1 V vs RHE in GDE configuration (details in the Supporting Information). Only selected molecules were studied with *in situ* XAS. Peroxide selectivity is referred to as high if above 2% in most of the potential region and low otherwise. ORR activity is referred to as high if the logarithm of the ORR current (in mAcm^–2^) is above 0.4 at 0.75 V vs RHE or low otherwise. Finally, the position on the volcano refers to the DFT-predicted *OH binding energy for the water containing (for 8EtFeP, 4PhFeP, 4NH2FeP) and water-free (for FePC, 4COOHFePC and 16ClFePC) molecules, compared to the optimal value of 0.86 eV.

Another difference between these groups is that, while for dual-peak molecules, the oxygen reduction current closely follows the voltammetric peak previously attributed to *OH desorption, single peak molecules display an oxygen reduction current at potentials hundreds of mV more positive than this redox peak. In this potential region, we would a priori expect Fe to be poisoned with *OH and hence to be inactive. Moreover, while DFT calculations predict the ORR activity of dual-peak molecules well, they fail to predict the performance of single peak molecules, further suggesting the existence of different mechanisms for these two groups.

In light of all of the experimental observations collected, we proposed three possible mechanisms for oxygen reduction on single-peak Fe macrocycles. The first mechanism requires the first step to be an outer-sphere electron transfer; the second mechanism involves an *OH axial ligand, and finally, the last mechanism implicates a potential-dependent *OH desorption in the absence of an electron transfer. Although all three mechanisms are identified as plausible, the last one would explain all of the experimental observations. For example, the change in the Tafel slope under this scenario would be caused by the gradual *OH desorption. Moreover, the presence of water as a back-side axial ligand would explain the higher peroxide yield and the XAS spectra.

More experimental evidence would be needed to unequivocally identify the mechanism of oxygen reduction on these macromolecules. In this sense, we envision that *in situ* spectroscopy measurements, such as surface enhanced infrared and Raman spectroscopy, will be crucial to gain further insight. However, this approach would also have its own challenges. For example, observing back-side adsorbed water would be difficult as this signal would overlap with the potential-dependent signals from interfacial and non-hydrogen bonded water. On the other hand, isotopically labeled experiments could be useful to elucidate the nature of the rate-determining step, in particular the involvement of a proton transfer.

In summary, although more *operando* characterization would be required to confirm the mechanism of ORR on single-peak molecules, all of the possibilities point to the existence of an oxygenated axial ligand. The presence of this ligand appears to control not only the number of redox peaks in the cyclic voltammogram but also the catalytic activity and selectivity. As a result, Fe macrocycles with this axial ligand display stronger *OH binding energies, lower ORR performance, and higher peroxide production, compared to those without it. These findings shine new light on what controls the activity of FeN_4_ active sites and pave the way to a more rational development of FeN_4_ catalysts. More generally, our work points to the overlooked role of axial ligands, which is a unique feature of single metal catalysts, as opposed to metal surfaces.

## Materials and Methods

### Materials

All of the macromolecules used in this work were purchased from Porphychem and used as received: Iron(II) phthalocyanine (FePC), Iron(II) 1,8,15,22-tetra(amino)phthalocyanine (4NH2FePC), Iron(II) 2,9,16,23-tetra(carboxy)phthalocyanine (4COOHFePC), Iron(II) 1,2,3,4,8,9,10,11,15,16,17,18,22,23,24,25 hexadeca(chloro)phthalocyanine (16ClFePC), Iron(III) 2,3,7,8,12,13,17,18 (octaethyl)porphyrin chloride (8EtFeP), Iron(II) 5,10,15,20 tetraphenyl,21*H*,23*H* porphyrin chloride (4PhFeP), 2,3,7,8,12,13,17,18 (octaethyl)porphyrin (8EtP), and 5,10,15,20 tetraphenyl,21*H*,23*H* porphyrin (4PhP). Thermally exfoliated graphene was used as a support and used as received.

### Synthesis of Fe-DCDA/G and DCDA/G

To test the ability of square wave voltammetry to identify peaks otherwise invisible in CV, we synthesised a pyrolyzed FeN4 catalyst following a procedure previously developed in the group. 4 g of dicyanamide was dispersed in 40 mL of water at 30 °C and stirred for 20 min; 400 mg of FeCl_3_ was then added to the solution and stirred for an additional 20 min. The temperature was then slowly increased to 100 °C, and the solution was left to dry for 4 h. The so-obtained solid (labeled Fe-DCDA) was further dried under vacuum at 80 °C for 24 h. Subsequently, Fe-DCDA was ground in a mortar with graphene in a weight ratio of 2:3 = Fe-DCDA:G. The product was pyrolyzed under nitrogen atmosphere at 550 °C for 4 h, using a heating rate of 3°C min^–1^, followed by two acid washing cycles in 0.1 M HClO_4_. The final product was collected by freeze-drying and labelled Fe-DCDA/G. DCDA/G was obtained following the same procedure in the absence of the iron precursor.

### Electrochemical Testing

In the synthesis of a typical catalyst, the iron macrocycles and graphene were ground in a mortar in a 1:1 mass ratio. The resulting graphene-supported macrocycles were then used to prepare the inks, composed by 2 mg of catalyst in 1 mL of 30%IPA in water with 18μL of Nafion. The solution was dispersed by 20 min of bath sonication and 10 min of probe ultrasonication.

The catalyst ink was spin-coated on a rotating disc electrode (3 mm radius glassy carbon RDE or 5 mm glassy carbon RRDE, Metrohm, previously polished with a micropolish cloth and 0.05μm alumina suspension) to obtain a loading of 0.14 mg/cm^2^. The electrodes were left to dry for 2 h before testing.

Electrochemical measurements were performed in a 3-electrode electrochemical cell, featuring a rotating disk working electrode, a graphite rod counter electrode, and a Hg/HgO reference electrode. The reference electrode was calibrated regularly, and all the potentials in this paper are reported vs RHE. The calibration was performed in H_2_-saturated 0.1 M KOH using a platinum RDE working electrode (from Metrohm) at 1600 rpm.

In a typical RDE experiment, the electrolyte (0.1 M KOH, Suprapur, Merch) was saturated with oxygen (^3^99.9998% Ultrapure Plus, Air Products), and linear sweep voltammograms (LSV) were collected at a scan rate of 10 mVs^–1^ and rotational speed ranging from 400 to 2400 rpm. Cyclic voltammograms (CVs) were collected in static, N_2_-saturated (^3^99.99998% BIP Plus, Air Products) 0.1 M KOH at a scan rate of 50 mVs^–1^. Finally, square-wave voltammograms (SWVs) were collected under the same conditions, using a potential step of 4 mV, modulation amplitude of 20 mV, and a frequency of 2 Hz, resulting in a scan rate of 8 mVs^–1^.

For the study of pH effects, the electrolyte concentration was varied at 0.01 M KOH, 0.1 M KOH, 1 M KOH, and 4 M KOH.

RRDE measurements were performed in 0.1 M KOH (Suprapur, Merch) oxygen-saturated electrolyte. The ring and disc currents were recorded while performing linear sweep voltammetry measurements at a scan rate of 10 mVs^–1^ with rotational speeds ranging from 400 to 2400 rpm. For all the measurements, the ring potential was set to 1.3 V vs RHE. The peroxide yield was found to have a low dependence on the rotation rate, and in this manuscript, we reported the results obtained at 1600 rpm. The peroxide yield was calculated according to the equation:
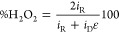
where *i*_R_ and *i*_D_ are current measured at the ring and disc, respectively. The collection efficiency, ε, was calibrated using the reversible ferrocyanide/ferricyanide reaction. The same electrodes as those used for RRDE measurements were used in the calibration. The electrode was rotated at speeds ranging from 400 to 2400 rpm in nitrogen-saturated electrolyte composed of 0.1 M KOH and 10 mM K_3_Fe(CN)_6_. Linear sweep voltammograms were collected at 10 mVs^–1^, varying the disc potential between 1.4 V vs RHE and 0.8 V vs RHE, while maintaining the ring potential at 1.9 V vs RHE. The ring current was corrected by subtracting the value measured at 0.4 V vs RHE (*i*_R0_), which is generated from the oxidation of species other than reduced Fe(CN)_6_^4–^, such as water, free Fe(CN)_6_^4–^, and impurities. The collection efficiency was then determined according to equation below and was found to be independent of rotational rate. For all the catalysts tested, the collection efficiency was determined to be 23 ± 0.5%
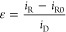


### DFT

All calculations were carried out using RPBE functional under Atomistic Simulation Environment (ASE) (a) with the GPAW code; (b) applying finite-difference approximation. We set unit k-points in three orthogonal directions, a grid-spacing of 0.18, and a minimum 3 Å vacuum. The geometry optimizations were performed with spin polarization and relaxation on, and the convergence criterion is satisfied once the force is below 0.05 eV Å^–1^. The results were finally corrected using a least-squares approach to correct for the fact that the values were calculated in a pure metal environment. All the DFT results plotted in this manuscript refer to the least-squares corrected data.

The equations used to calculate the binding energies are listed Section S4, and the results are summarized in Tables S1–S3.

To simulate the XAS spectra, we used half core-hole in 1s (hch1s) setup for the Fe atom. We calculated the total energy difference of the hch1s first core excited state and its ground state as the absolute energy scale. For spectrum generation, we applied the Haydock recursion method. More details are provided Section S4.

### *In Situ* XAS Measurements

Operando XAS measurements were performed at room temperature using an in-house-designed gas-diffusion-electrode cell placed 45° to the incident beam. The operando data were collected as HERFD XANES, at I20-scanning, Diamond light source, using a Si(111) monochromator and spherically bent Ge(440) crystal analyzers aligned to the Fe Kα line in combination with the available 4 element medipix detector. Details of the in operando cell and of the experimental conditions can be found in Section S3.

### Water Solubility and Porosimetry Analysis

Nitrogen sorption isotherms were measured at 77 K using a Tristar sorption analyzer from Micromeritics. Before the measurements, the samples were degassed at 453 K for 24 h in vacuum. The surface area was determined from the linearized BET equation; the micropore volume was obtained via the t-plot, and the pore volume distribution was determined using the NLDFT model for carbon slit pores.

Water sorption isotherms were measured at 298 K, using a 3flex sorption analyzer from Micromeritics. Before the measurements, the samples were degassed at 453 K for 24 h in vacuum, and water was degassed by performing three freeze-thaw cycles.

## Supporting Information Available

Additional electrochemical measurements (section S1), details regarding the derivation of the Tafel analysis and simulation of the Tafel slope (section S2), nitrogen-sorption isotherms and pore size distribution, experimental details, and results of operando XAS (section S3), and additional details regarding assumptions, parameters, and results for DFT simulations (section S4) (PDF)
