# Supplementary material for: Same FeN4 Active Site, Different Activity: How Redox Peaks Control Oxygen Reduction on Fe Macrocycles
Source: ACS Electrochem. 2025 Jan 8;1(5):617–32. doi: 10.1021/acselectrochem.4c00146 (PMC12051206; doi:10.1021/acselectrochem.4c00146)
Supplement: Supplementary file 1 — ec4c00146_si_001.pdf [file ec4c00146_si_001.pdf]

Supporting Information

Same FeN4 active site, different activity – How redox peaks control oxygen reduction on Fe macrocycles

Silvia Favero[a]*, Ruixuan Chen[a], Joyce Cheung[a], Luke Higgins[b], Hui Luo[c], Mengnan Wang[a], Jesus Barrio [a], Maria Magdalena Titirici[a], Alexander Bagger[d]*, Ifan E. L. Stephens[e]*

[a] Department of Chemical Engineering, Imperial College London, South Kensington Campus, SW7 2AZ, London, UK
[b] Diamond Light Source, Didcot OX11 0DE, UK
[c] Institute for Sustainability, University of Surrey, Surrey GU2 7XH, UK
[d] Department of Physics, Danish Technical University, 2800 Kongens Lyngby, Denmark. Email: alexbag@dtu.dk
[e] Department of Materials, Imperial College London, South Kensington Campus, SW7 2AZ, London, UK. Email: i.stephens@imperial.ac.uk

Table of content

- **Section S1: Electrochemistry**
  - Fig. S1: CV of the Fe macrocycles in N2
  - Fig. S2: CV and SWV of the heat-treated FeDCDA/G and DCDA/G
  - Fig. S3: SWV study of pH effect
  - Fig. S4: RRDE and peroxide efficiency results
- **Section S2: Tafel Analysis**
  - Derivation of a micro-kinetics analysis
  - Calculation of the kinetic current via KL equation and KL plot
  - Fig. S5: Simulation of *OH coverage, Tafel plot and Tafel slope
  - Fig. S6: Experimental Tafel plot
- **Section S2: Characterization**
  - Fig S7: N_2-sorption isotherm and pore size distribution
- **Section S3: *Operando* XAS**
  - Experimental details of operando XAS
  - Fig. S8: Custom-built in-situ electrochemical cell for XAS measurements
  - Fig. S9: Experimental results of *operando* XAS
  - Fig. S10: XAS spectra simulation results
- **Section S4: DFT simulations**
  - Details of DFT calculations and assumptions
  - Table S1: Results of DFT-calculated binding energies
  - Fig. S11: Comparison of experimental (Tafel plot) and DFT-predicted high potential peak position
  - Fig. S12: Volcano plots comparing DFT and experimental results
  - Fig. S13: Scaling relationship between DFT calculated binding energies
  - Table S2: Scaling relationship between DFT calculated binding energies

Section 1. Electrochemistry

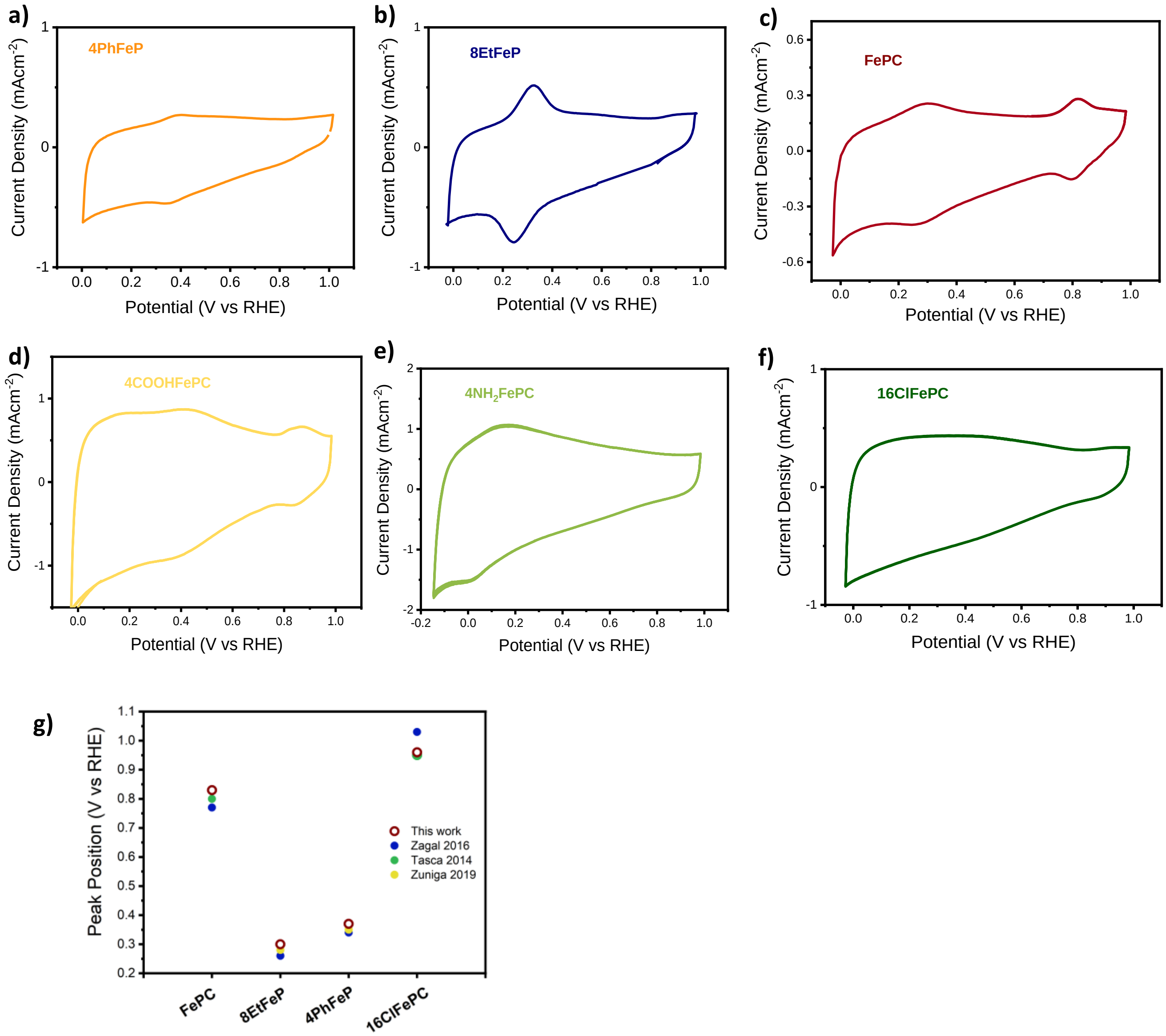

Figure S1 Cyclic voltammogram of the selected macromolecules. Data were recorded in nitrogen saturated 0.1M KOH, at a scan rate of 50mV/s. The results are shown for a) 4PhFeP, b) 8EtFeP, c) FePC, d) 4COOHFePC, e) 4NH2FePC, f) 16ClFePC

g) comparison of peak position reported here with literature results: Zagal 2016[5], Tasca 2014[6], Zuniga 2019[7]

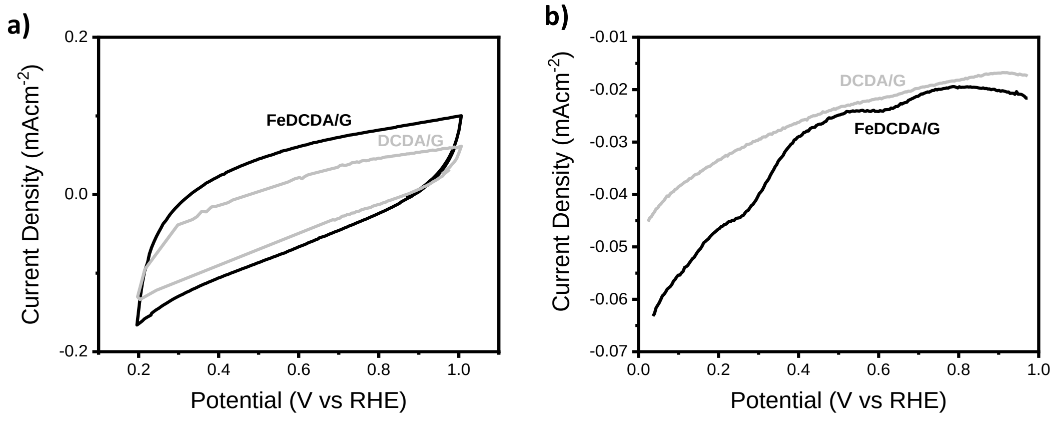

Figure S2 a) cyclic voltammogram of FeDCDA/G and DCDA/G, recorded in N_2-saturated 0.1M KOH, with a scan rate of $50mVs^{-1}$.
b) Square wave voltammogram of FeDCDA/G and DCDA/G, recorded in N_2-saturated 0.1M KOH, using a potential step of 4mv, modulation amplitude of 20mV and a frequency of 2Hz, resulting in a scan-rate of $8mVs^{-1}$

To test the ability of this technique to identify redox peaks, we performed SWV on a pyrolyzed FeN4 catalyst (labelled FeDCDA/G) previously synthesised in our group, for which no peaks could be distinguished in the cyclic voltammograms (Figure S2a). Surprisingly, two peaks were instead clearly visible in the square-wave voltammograms (Figure S2b), and control experiments in absence of iron confirmed that the peaks originated from electron transfers at the Fe centre. We further noticed that low frequencies offered the highest peak resolution, with 2Hz presenting the optimal balance between peak resolution, time consumption and signal-to-noise ratio.

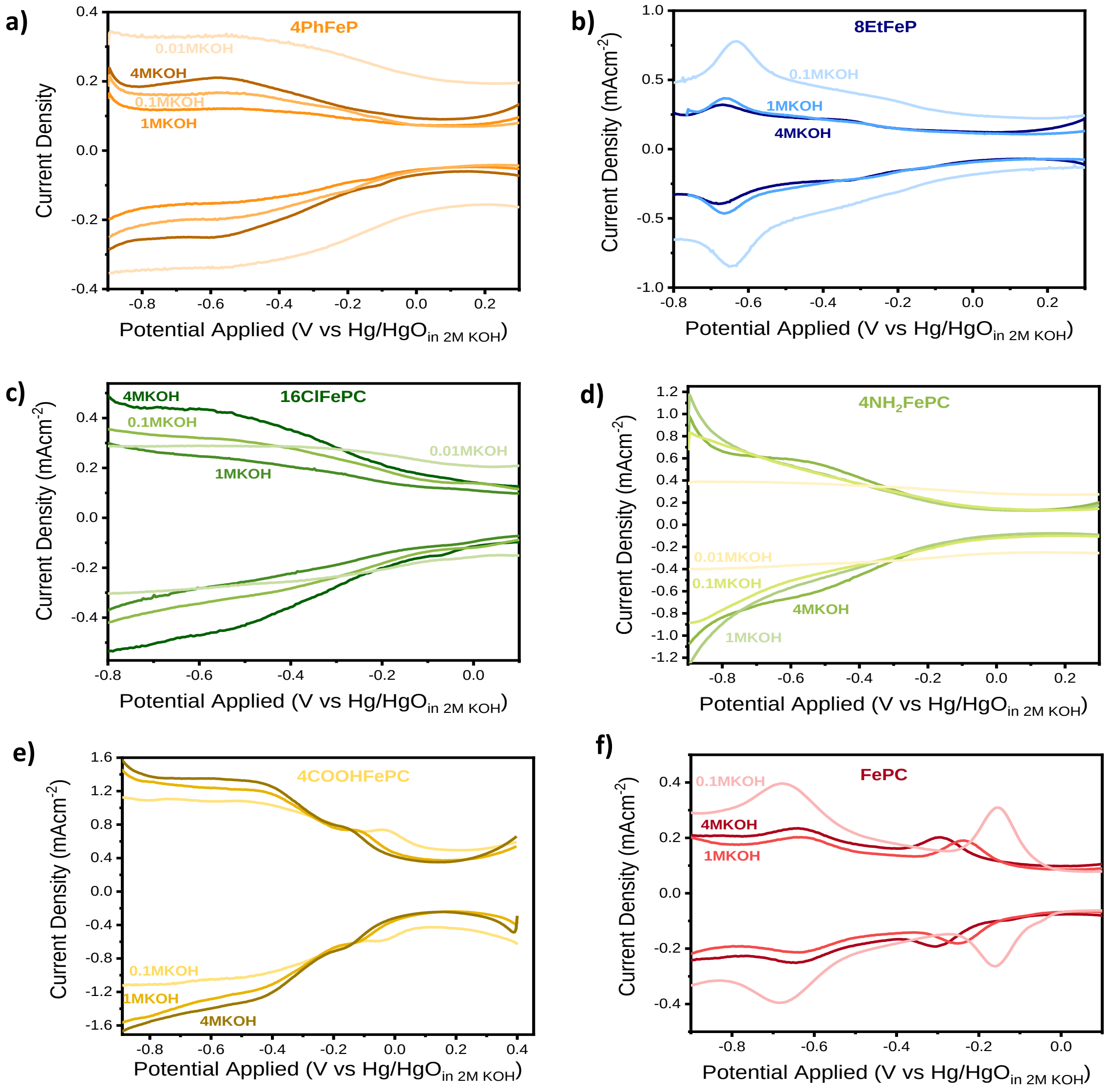

Figure S3 Square-wave voltammograms of iron macrocycles at different pH, from the darker to the lighter voltammogram data were collected in 4M KOH, 1M KOH, 0.1M KOH and 0.01M KOH. Data were collected in static conditions, using a potential step of 4mv, modulation amplitude of 20mV and a frequency of 2Hz, resulting in a scan-rate of 8mVs^{-1}. Results are shown for a) 4PhFeP, b) 8EtFeP, c) 16ClFePC, d) 4NH2FePC, e) 4COOHFePC, f) FePC

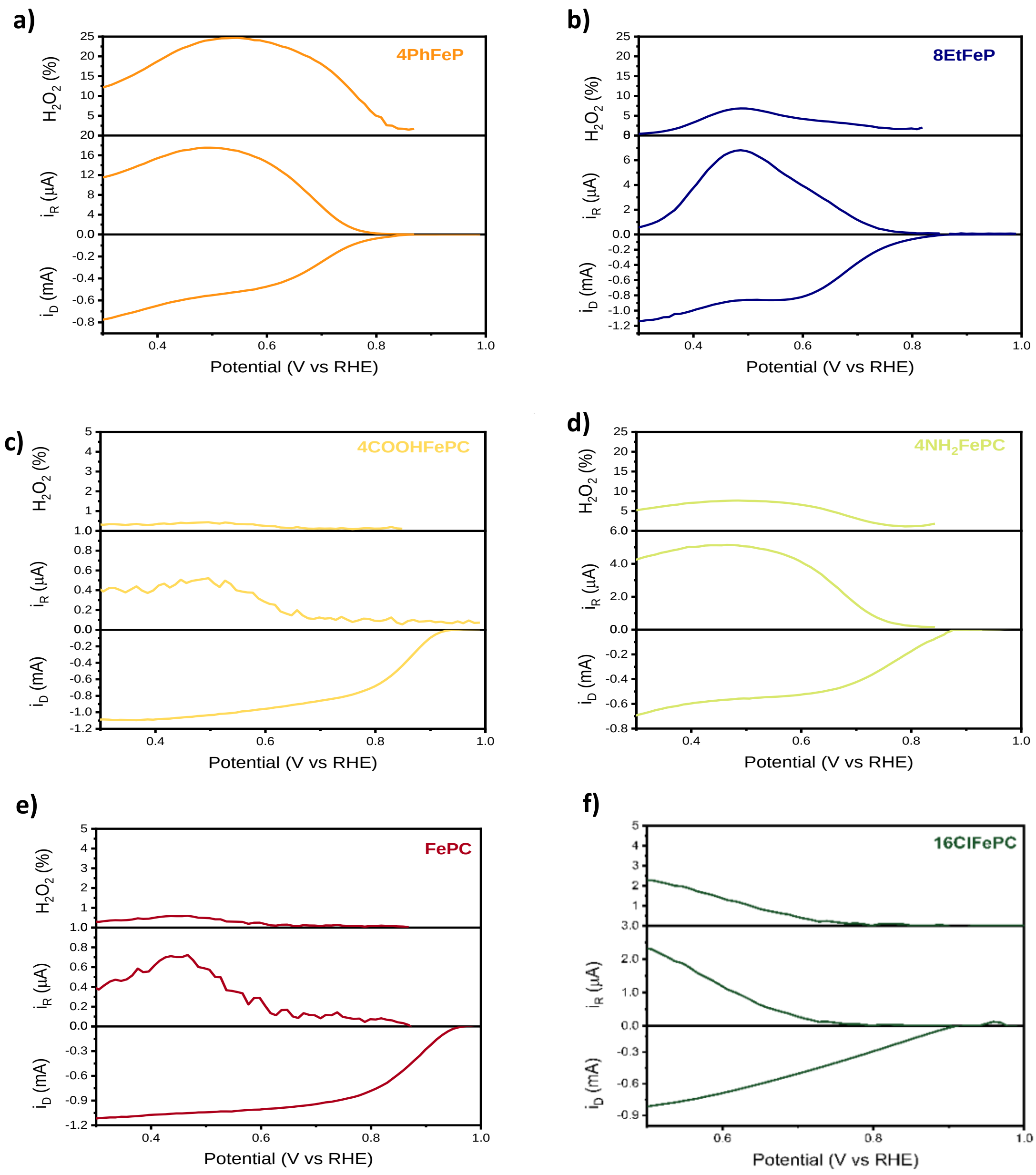

Figure S4 RRDE results of the selected macromolecules. Each plot shows from top to bottom, the peroxide yield, the current measured at the ring and the current measured at the disc. Data were obtained in oxygen saturated 0.1M KOH, with a 5mm rotating ring-disc electrode, at a rotational speed of 1600 rpm. Data are shown for a) 4PhFeP, b) 8EtFeP, c) 4COOHFePC, d) 4NH2FePC, e) FePC, f) 16ClFePC

Discussion on the Tafel Slope

The dependence of oxygen reduction kinetics on potential, are often evaluated using the Tafel slope. This is defined as the change in potential necessary to increase the kinetics current by an order of magnitude. The experimentally determined Tafel slope depends on the rate determining step and can be compared with theoretically derived values, obtained assuming different rate-determining steps. Since this process can be complicated, the surface coverage of intermediates is often assumed as constant, generally postulating complete coverage (θ=1) of a single species. However, intermediates coverage is likely to change with potential, and this approach can lead to an incorrect identification of the rate-determining step. A recent work by Takanabe and co-workers, thoroughly analyzed the expected Tafel slope, depending on the assumed rate-determining step, for aqueous electrocatalytic reactions, including oxygen reduction, showing how changes in the surface coverage of intermediates can lead to changes in the Tafel slope.[1]

In this work, we will derive a coverage-dependent expression of the Tafel slope, which matches the experimentally observed values. However, we would like to highlight that multiple rate determining steps could lead to the same Tafel slope preditctions.

From the Butler-Volmer equation, the current given a single rate-limiting potential-dependent step is given by:

$$j = j_0 \theta_{RDS} \exp(\alpha f \eta_{RDS})$$

Where j is the kinetic current density, j0 is the theoretical exchange current density, η_{RDS} is the overpotential, i.e. the difference between the applied potential and the thermodynamics potential for the RDS, θ_{RDS} is the coverage of the rate-limiting species. α is the simmetry factor of the rate limiting step, which for a single electron transfer can be assumed to be 0.5, and f=F/RT, where F is the Faraday constant, R is the gas constant and T is the temperature.. For the case where the coverage of the rate-determining step is independent of potential than

$$j \propto \exp(\alpha f \eta_{RDS})$$

the Tafel slope can be calculated as:

$$Tafel\ Slope = \frac{\eta}{\log j} = \frac{\ln(10)}{f}\frac{1}{\alpha} = 120\ mV/dec$$

Kowing that ln(10)/f has a value of 60mV/dec and assuming a simmetry factor of 0.5, this leads to the well-known conculsion that if the reaction is limited by a single electron transfer, the Tafel slope is 120mV/dec. This is in-line with the experimentally observed value of 120 mV/dec, at high overpotential, which suggests that the reaction at high overpotential is limited by a single electron transfer. However, at lower overpotential, a value of 40mV/dec is observed.

Derivation of a micro-kinetics model

Changes in the Tafel slope result from changes in rate-determining step with potential, superposistion of different sites contributing to the overall activity or changes in the coverage of the rate limiting species.

Here we assume that the reaction mechanism does not change with potential, which is a common assumption in microkinetics models.[1–3] Additionally, a change in mechanism with static coverage would not explain the observed Tafel slope of 40mV/dec at low overpotential, as the mechanism at

low overpotential could either include an electron-transfer rds, which would lead to a Tafel slope of 120mV/dec, or a chemical step, which would lead to an infinite slope.

The case of multiple sites contributing to the activity can also not explain the experimental observations. In fact, as Soren et al. showed, the superimposition of two sites can only lead to a reduction of the Tafel slope.[3]

Finally, we consider the case where the coverage of the rate-limiting species is not constant. In particular, DFT calculations and experimental observations of the cyclic voltammetry peaks suggest that the the potential-determining step is the desorption of the *OH, which blocks the active site and that the reaction at low overpotential might be limited by the coverage of adsorbed hydroxiles. At higher overpotentials, a later step, such as oxygen adsorption, could limit the reaction rate, as suggested by Koper and co-workers.[4]

This mechanism would be represented by the following equations:

$$FeOH + e^- \leftrightarrow Fe + OH^- \qquad 1)$$

$$Fe + O_2 + e^- \leftrightarrow FeO_2^- \qquad 2)$$

If we assumed the second step to be rate-determining, then the first step should be at equilibrium, which means that the forward a backward reaction rates (r_1, r_{-1}) for step 1 are equal.

$$r_1 = k_{1,0}\exp(-\alpha_1 f\eta_1)\,\theta_{FeOH} = r_{-1} = k_{-1,0}\exp(\alpha_1 f\eta_1)\,\theta_{Fe}[OH^-]$$

If 2) is the RDS, it is alsoo possible to assume that the only species present are Fe and FeOH, therefore

$$\theta_{FeOH} + \theta_{Fe} = 1$$

Substituing this in the equation above and isolating θ_{Fe} we obtain:

$$k_{1,0}\exp(-\alpha_1 f\eta_1)\,(1-\theta_{Fe}) = r_{-1} = k_{-1,0}\exp(\alpha_1 f\eta_1)\,\theta_{Fe}[OH^-]$$

$$k_{1,0}\exp(-\alpha_1 f\eta_1) = \left(k_{-1,0}\exp(\alpha_1 f\eta_1)\,[OH^-] + k_{1,0}\exp(-\alpha_1 f\eta_1)\right)\theta_{Fe}$$

$$\theta_{Fe} = \frac{k_{1,0}\exp(-\alpha_1 f\eta_1)}{k_{-1,0}\exp(\alpha_1 f\eta_1)\,[OH^-] + k_{1,0}\exp(-\alpha_1 f\eta_1)} = \frac{K_{1,0}\exp(-f\eta_1)}{[OH^-] + K_{1,0}\exp(-f\eta_1)}$$

Where $K_{1,0} = \frac{k_{1,0}}{k_{-1,0}}$ and $\alpha_1 = 0.5$

This gives us an expression for the coverage of the empty Fe site, as a function of potential, which predicts a increase in coverage from 0 to 1, as we go from high potentials, where the surface is poisoned with OH, to low potentials, where the active sites are empty.

$$\theta_{Fe} = \frac{K_{1,0}\exp(-f\eta_1)}{[OH^-] + K_{1,0}\exp(-f\eta_1)}$$

Now, assuming that step 2 is the rate-limiting step, the reaction rate can be calculated according to:

$$r_2 = k_{2,0}\,\theta_{Fe}\exp(-\alpha_2 f\eta_2)P_{O2}$$

Substituting the expression for the coverage, we obtain:

$$\boldsymbol{r_2 = k_{2,0}\exp(-\alpha_2 f\eta_2)\frac{K_{1,0}\exp(-f\eta_1)}{[OH^-] + K_{1,0}\exp(-f\eta_1)}P_{O2}}$$

This is the rate expression, valid over the whole potential range.

At high overpotentials

Now let's look at high overpotentials. In this case $\eta_1 \gg 0$, from which, as expected the coverage of θ_{Fe} becomes close to 1

$$\theta_{Fe} = \frac{K_{1,0}\exp(-f\eta_1)}{[OH^-] + K_{1,0}\exp(-f\eta_1)} = \frac{1}{\frac{[OH^-]}{K_{1,0}\exp(-f\eta_1)} + 1} \sim 1$$

Therefore the rate expression can be simplyfied to

$$r_2 \sim k_{2,0}\exp(-\alpha_2 f\eta_2)\, P_{O2}$$

From which the Tafel slope becomes equal to the expression derived above for a single electron transfer

$$\ln(10)\log\left(r_2/k_{2,0}P_{O2}\right) = -\alpha_2 f\eta_2 \quad \rightarrow \eta_2 \propto \frac{\ln(10)}{f\alpha_2}\log(i)$$

$$Tafel\ Slope = \frac{\eta}{\log j} = \frac{\ln(10)}{f}\frac{1}{\alpha_2} = 120\ mV/dec$$

Therefore, **the predicted Tafel slope at high overpotential is 120mV/dec**

At low overpotentials

In the case of low overpotentials, the coverage is a function of potential and cannot be simplified.

Therefore the Tafel slope becomes:

$$i \propto \exp(-\alpha_2 f\eta_2)\frac{K_{1,0}\exp(-f\eta_1)}{[OH^-] + K_{1,0}\exp(-f\eta_1)}$$

$$\log i \propto \log\left(\exp(-\alpha_2 f\eta_2)\right) \quad + \log\left(\frac{K_{1,0}\,exp(-f\eta_1)}{[OH^-] + K_{1,0}\,exp(-f\eta_1)}\right)$$

If the overpotential is low $\eta_1 \ll 0$, the expression can be simplified to

$$\begin{aligned}\log i \propto -\alpha_2 f\eta_2 - \log\left(\frac{K_{1,0}\,exp(-f\eta_1) + [OH^-]}{K_{1,0}\,exp(-f\eta_1)}\right) \\ = -\alpha_2 f\eta_2 - \log\left(1 + \frac{[OH^-]}{K_{1,0}\,exp(-f\eta_1)}\right) \sim -\alpha_2 f\eta_2 - \log\left(\frac{[OH^-]}{K_{1,0}\,exp(-f\eta_1)}\right) \\ = -\alpha_2 f\eta_2 - f\eta_1 - \log\left(\frac{[OH^-]}{K_{1,0}}\right)\end{aligned}$$

$$Tafel\ Slope = \frac{\eta}{\log j} = \frac{\ln(10)}{f}\frac{1}{\alpha_2 + 1} = 40\ mV/dec$$

Therefore, **the predicted Tafel slope at high overpotential is 40mV/dec**

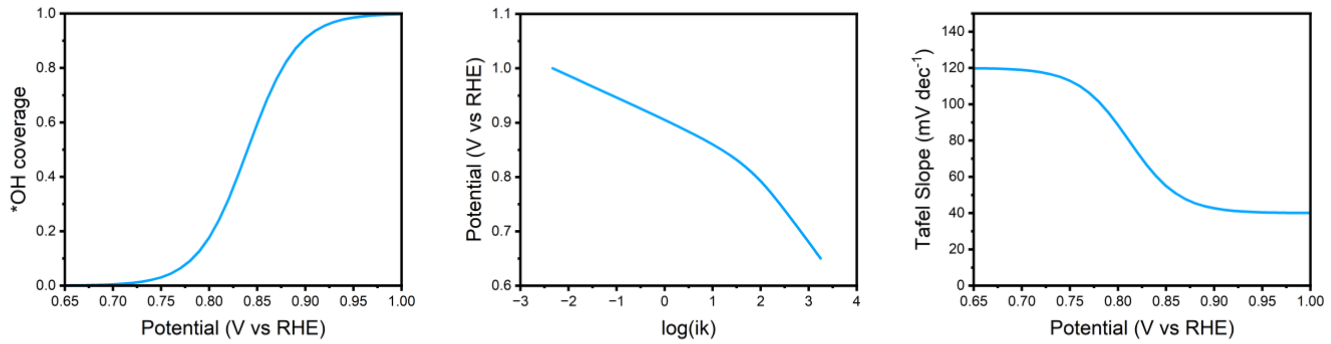

Figure S5: Simulated OH* coverage, as a function of the applied potential, Tafel plot and Tafel slope as a function of potential. Graphs obtained using the equations described above, and assuming the following: η_1=V-0.9eV, η_1=V-0.8eV, $K_{1.0}$=1, $k_{2,0}$=100, $[OH^-]$=0.1. Notice that the value of $k_{2,0}$ has no influence over the first and third plot, or on the shape of the second plot, it only influence the magnitude of ik. On the contrary, all the other values have no influence on the shape, but have the only effect of shifting the curve on higher or lower potential.

If this analysis is correct, the change in Tafel slope should correspond to a change in coverage of the *OH species and a Tafel slope of 120mV/dec should be reached when the *OH coverage is 0. As expected, for the case of two peak molecules, the change in Tafel slope also happens at the same potential as the high-potential CV peak, which is attributed to *OH desorption. This means that the change in Tafel slope can also be used to monitor *OH desorption in one-peak molecules.

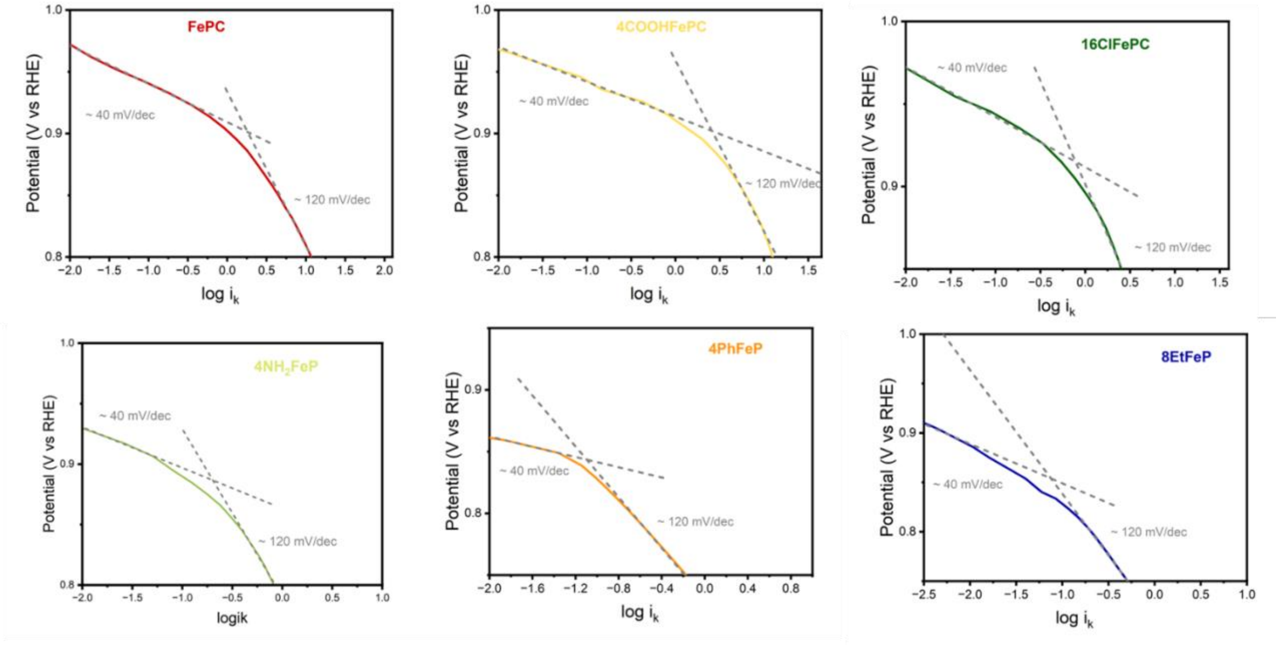

Figure S6 Tafel plot of the selected single peak macrocycles. On the x-axis is the logarithm of the kinetic current density (in mAcm-2). The kinetic current density was extracted from the measured current density (i) using the Koutecky-Levich equation: ik=(I^-1-id^-1)^-1. Where id is the theorical diffusion-limited current density, as determined by the Levich equation. The dashed lines show the linear of high and low overpotential linear region of the Tafel plot. The value displayed on the graphs represent the potential at which the two linear fits intercept.

Section S2: Characterization

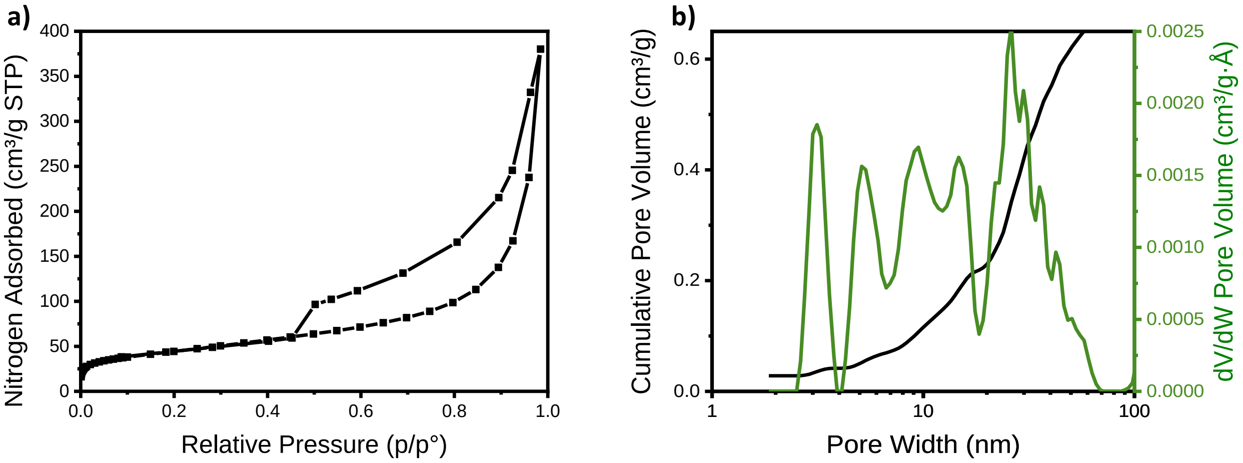

Figure S7 a) N_2 adsorption isotherm of FePC deposited on graphene and b) porosity distribution by NLDFT

Section S3 Operando X-ray adsorption spectroscopy

Experimental set-up

Operando XAS measurements were performed at room temperature using an in-house designed gas-diffusion-electrode cell placed at 45° to the incident beam (Figure S10). The cell is composed of a hydrophobic carbon paper, on which the catalyst was spray coated, to obtain a total catalyst loading of 1 mg cm^{-2}, corresponding to an iron loading of 0.05 mg cm^{-2}. On one side of the paper is a gas inlet, where oxygen or nitrogen was flowed, on the opposite side is the electrolyte (1 M KOH), containing a small reference electrode (Ag/AgCl) and counter electrode (platinum wire). Due to the presence of these electrodes, the electrolyte thickness cannot be reduced below 4 mm, therefore the measurements were performed in fluorescence mode.

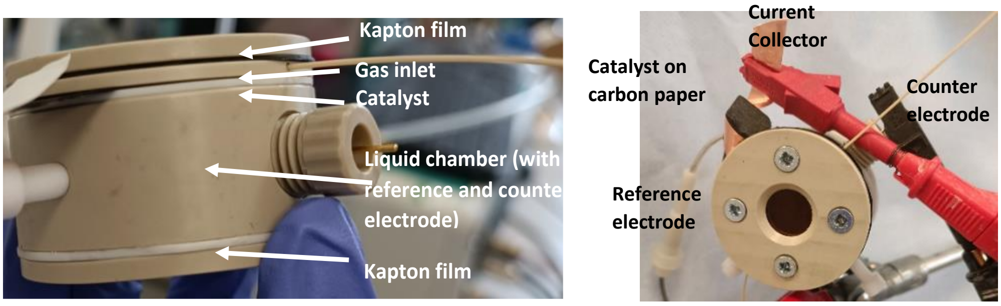

Figure S8 custom built GDE cell, for *in-situ* XAS measurements

Operando data were collected as high energy resolution fluorescence detection (HERFD) X-ray absorption near-edge spectroscopy (XANES), at I20-scanning (Diamond Light Source). The scanning branch of the beamline is equipped with an Si (111) scanning four bounce monochromator. The X-ray emission spectrometer utilised to collect HERFD measurements is composed of three 100 mm spherically bent Ge (440) striped crystal analysers operating in Johann configuration lying on the 1m Rowland circle. The analyser crystals were aligned to the Fe K_α line (6.404 KeV) and focussed onto a 4 element medipix area detector, HERFD spectra were collected by scanning the monochromator energy over the XANES region, with a fixed spectrometer energy. Four samples were tested *in-operando*: 4PhFeP/G, 8EtFeP/G, FePC and 4NH$_2$FePC/G. For the sample FePC/G extended X-ray absorption fine (EXAFS) was also collected, using a Vortex 4 element silicon dirft florescence detector. The EXAFS of 4 standards pellets (FeO, Fe2O3, Fe(II)PC and Fe(III)PC-Cl) were collected in transmission geometry using the available ionisation chamber with a 1:1 mix He to N_2. The data was normalised to the incident intensity and processed using the Athena software package.

Results

Figure S11 shows the results on *operando XAS* measurements, for selected iron macromolecules: FePC, 4PhFeP, 8EtFeP, 4NH$_2$FePC. FePC, which belongs to the group of molecules that show a high potential peak, shows a potential-dependent change in the HERFD XANES spectra under nitrogen (Figure S11a). Upon application of a reducing potential, the white line shifts to higher energy values, the whiteline intensity decreases, and the intensity of the pre-edge peak located at 7118 eV increases. On the other side, all the molecules belonging to the second group (4NH2FePC – Figure S11e, 4PhFeP – Figure S11g, 8EtFeP – Figure S11i) don't show any change in the HERFD XANES spectra upon application of a reducing potential while flowing nitrogen. On the contrary, these molecules showed a change when oxygen was flowing (Figure S11f, S11h, S11l), confirming that the iron centre is the active site.

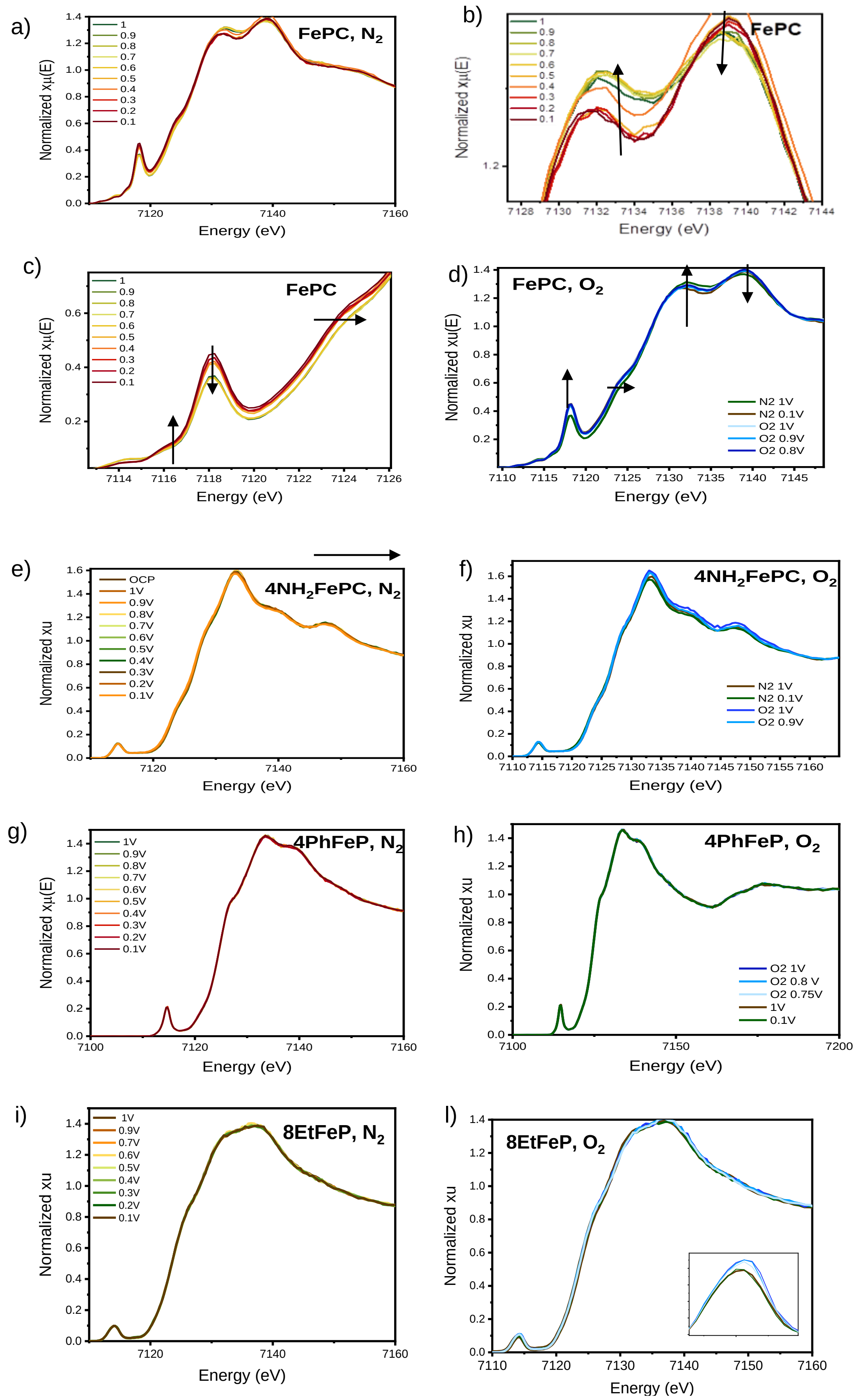

Figure S9: operando HERFD XANES of: a) FePC in N2-saturated electrolyte (b) and c) are zoom ins of the same spectra), d) FePC in O2-saturated electrolyte, e) 4NH2FePC in N2-saturated electrolyte, f) 4NH2FePC in O2-saturated electrolyte, g) 4PhFeP in N2-saturated electrolyte, h) 4PhFeP in O2-saturated electrolyte, i) 8EtFeP in N2-saturated electrolyte, l) 8EtFeP in O2-saturated electrolyte

To confirm that the absence of a spectral change in the nitrogen-saturated environment for the single-peak-molecules is the result of adsorbed water, we simulated the XANES spectra of the selected molecules, with and without adsorbed water. Figure S8 shows the results of the simulation, compared to the experimental data (lowest curve). As it can be observed for the case of 4PhFeP, the experimental data show a pre-edge at around 7115eV. According to our simulations, this peak is only present when water is adsorbed on the iron centre, either in the form of OH-Fe-H2O, or H2O-Fe-H2O. Similar observations were obtained for 8EtFeP, while the analysis for 4NH2FePC is inconclusive.

On the other side, FePC shows a pre-edge peak at a slightly higher energy of 7118eV. This feature is only present in our simulations, for Fe(II) and Fe(III), both of which do not have adsorbed water.

This analysis further supports our hypothesis that the two families of Fe macrocycles differ for the presence of adsorbed water.

Finally, we have previously mentioned that for the case of FePC, an oxygen reduction current is observed at slightly higher potential than the high-potential redox peak. We propose that this phenomenon is due to the presence of oxygen, which destabilizes the *OH intermediate, allowing OH desorption to happen at higher potential than in nitrogen.

If we accept that the XAS spectral change observed for the case FePC is a result of OH desorption, then the operando results confirm that OH desorption happen at higher potential in oxygen than in nitrogen-saturated electrolyte there is a gradual change in the XAS spectra happening at around 0.7V vs RHE, corresponding to the desorption of the *OH intermediate and showing a gradual reduction of the iron centre. However, for the same measurements done in oxygen (Figure S11d) the iron centre appears reduced already at 1V vs RHE, which is above the onset potential for oxygen reduction.

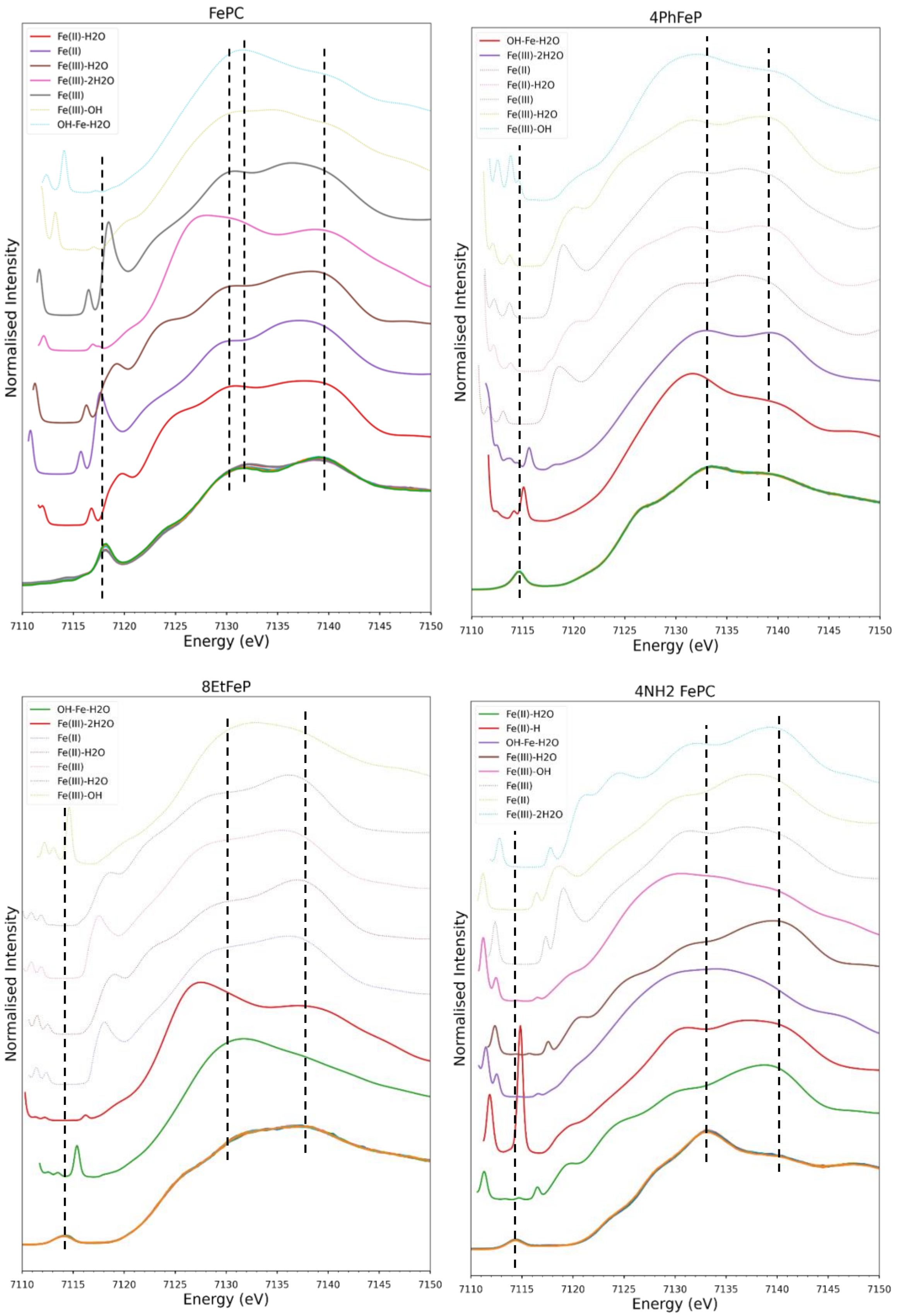

Figure S10: DFT-simulated spectra for the molecules a) FePC , b) 4PhFeP, c) 8EtFeP, d) 4NH2FePC with different adsorbed oxygenated species (the bottom spectra shows the experimental data for comparison).

Section S4 DFT

All calculations were carried out using RPBE functional under Atomistic Simulation Environment (ASE)[8] with the GPAW code[9,10] applying finite-difference approximation. We set unit k-points in three orthogonal directions, a grid-spacing of 0.18 and a vacuum of minimal 3Å. The geometry optimisations were performed with spin polarisation and relaxation on, and the convergence criterion is satisfied once the force is below 0.05 eV Å^{-1}.

The binding energies were calculated with the following three equations:

$$H_2O + * \longrightarrow *O + H_2$$

$$\Delta G(*O) = E(*O) - E(*) + E(H_2) - E(H_2O) + 0.05 \quad (1)$$

$$H_2O + * \rightarrow *OH + H^+ + e^-$$

$$\Delta G(*OH) = E(*OH) - E(*) + \frac{1}{2}E(H_2) - E(H_2O) + 0.35 - 0.3 \quad (2)$$

$$2H_2O + * \rightarrow *OOH + 3(H^+ + e^-)$$

$$\Delta G(*OOH) = E(*OOH) - E(*) + \frac{3}{2}E(H_2) - 2E(H_2O) + 0.4 - 0.3 \quad (3)$$

Where the positive constant represents the zero point energy corrections and entropic contributions to the free energies and the negative number incorporates the implicit water solvation into the system considering its stabilisation effect[11]. The calculated results were summarised in the following two tables where m is the spin relaxed magnetic moment that can be related to Fe^{2+} (m=0) and Fe^{3+} (m=2). First for m=0, considering its highly symmetric geometry structure and paired electrons, it is sensible to conclude that the valence is Fe^{2+}. As not only Fe^{4+} is too energy demanding to exist in our system, but also the spin relaxation is not compromised for the lowest energy optimisation purpose in a highly symmetric structure. Namely, m=0 is exact, it is not the approximation for m=0.5. On the flip side, this is not the case for m=2. And it is supported by both simulations and experiments. By adding an electron in previous charge neutral modelling system for m=2, we found that the m shifted from 2 to near 1.5 with the lowest absolute DFT energy had almost identical binding energies as m=2. It suggests that the compromised magnetic moment m=2 is effectively 1.5. Our proposed schemes are either Fe^{+}->Fe^{2+} or Fe^{2+}->Fe^{3+}, and m~1.5 can only be Fe^{3+} in accordance with experiment fittings.

We ran the simulations from m=0 and m=2 as the initial states and they converged to the same species *X (X= O, OH, OOH) without further variations in spin.

Energy (eV)	E(*)	E(*O)	E(*OH)	E(*OOH)	G(*O)	G(*OH)	G(*OOH)
4PhFeP(m=0)	-520.05					0.22	
4PhFeP(m=2)	-520.60	-526.51	-530.81	-535.19	1.72	0.77	4.04
AABB_8EtFeP(m=0)	-512.39					0.31	
AABB_8EtFeP(m=2)	-512.93	-518.87	-523.07	-527.63	1.70	0.85	3.93

ABAB_8EtFeP(m=0)	-512.40					0.33	
ABAB_8EtFeP(m=2)	-512.94	-518.86	-523.05	-527.56	1.73	0.88	4.02
FePC (m=0)	-407.22					0.44	
FePC(m=2)	-407.72	-413.45	-417.76	-422.15	1.91	0.95	4.20
16ClFePC (m=0)	-378.21					0.50	
16ClFePC (m=2)	-378.69	-384.36	-388.70	-393.13	1.96	0.98	4.19
$4NH_2FePC$ (m=0)	-454.99					0.43	
$4NH_2FePC$ (m=2)	-455.44	-461.22	-465.55	-469.95	1.86	0.89	4.12
4COOHFePC(m=0)	-497.75					0.48	
4COOHFePC (m=2)	-498.25	-503.94	-508.26	-512.70	1.95	0.99	4.19

Table S1 DFT Energies (E) and Binding Energies (G) of oxygenated ORR intermediates species (*O, *OH, *OOH) on the selected iron macrocylcles, for spin relaxed magnetic moments m=0 and m=2,

The results so far were calculated without taking into account the solvation effects. The results for m-0 without any solvation correction match the experimental results quite well, but those for m=2 don't. A first approach to correct for the solvation effect is to take the value previously reported for a platinum surface (0.3eV). Finally, we also considered the case of 0.45eV solvation effect, which would allow a correct prediction of the FePC *OH desorption peak position.

The results without solvation and for solvation of 0.3eV and 0.45eV are plotted in Figure S11

DFT results

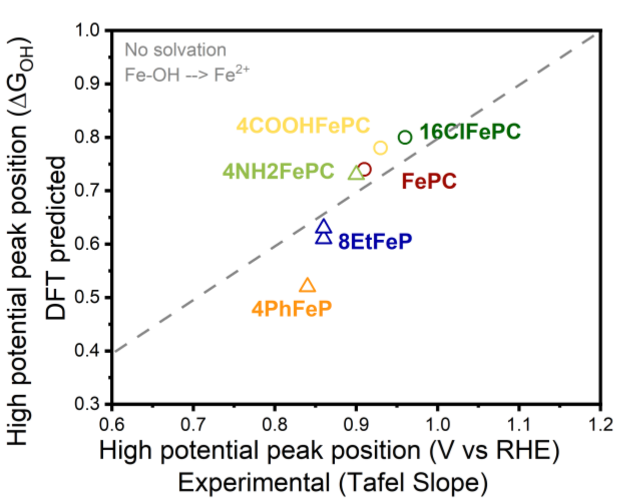

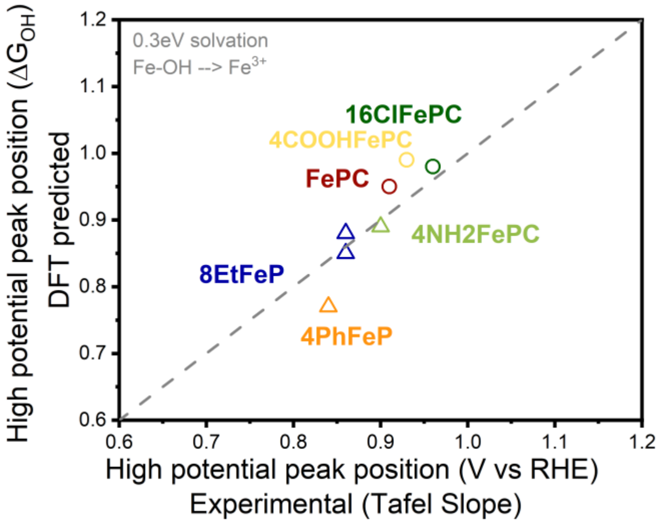

Figure S11: a) Comparison of the experimental and DFT predicted high potential peak. The experimental value is derived from the Tafel plot analysis, while the DFT prediction are for the case of OH desorption, with a change in oxidation state to 2+, without solvation

b) Comparison of the experimental and DFT predicted high potential peak. The experimental value is derived from the Tafel plot analysis, while the DFT prediction are for the case of OH desorption, without a change in oxidation state with 0.45eV solvation

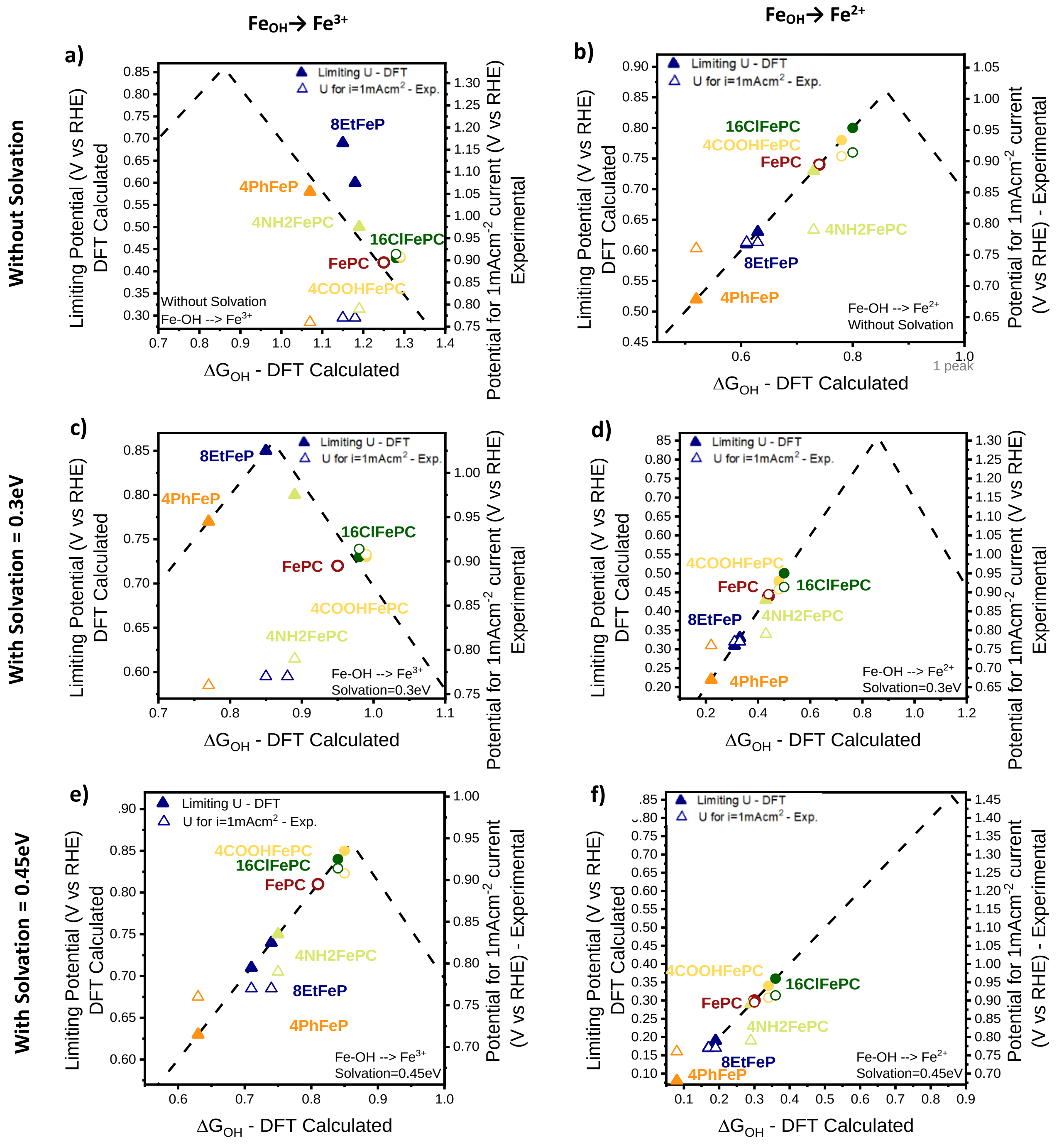

Figure S12: Volcano plot showing on the left y-axis the DFT- limiting potential (full symbols) for oxygen reduction and on the right y-axis the experimentally determined potential necessary to reach a current density of 1mAcm-2. Both data set are plotted as a function of the DFT-predicted *OH binding energy. The experimental data are scaled to fit the prediction for FePC.

The DFT calculations are obtained assuming that

a) OH desorption happens without a change in Fe oxidation state: Fe-OH to Fe^{3+} and omitting solvation corrections

b) OH desorption happens with a change in Fe oxidation state: Fe-OH to Fe^{2+} and omitting solvation corrections.

c) OH desorption happens without a change in Fe oxidation state: Fe-OH to Fe^{3+} and with 0.3eV solvation corrections
d) OH desorption happens with a change in Fe oxidation state: Fe-OH to Fe^{2+} and with 0.3eV solvation corrections.
e) OH desorption happens without a change in Fe oxidation state: Fe-OH to Fe^{3+} and with 0.45eV solvation corrections
f) OH desorption happens with a change in Fe oxidation state: Fe-OH to Fe^{2+} and with 0.45eV solvation corrections.

Scaling Relation

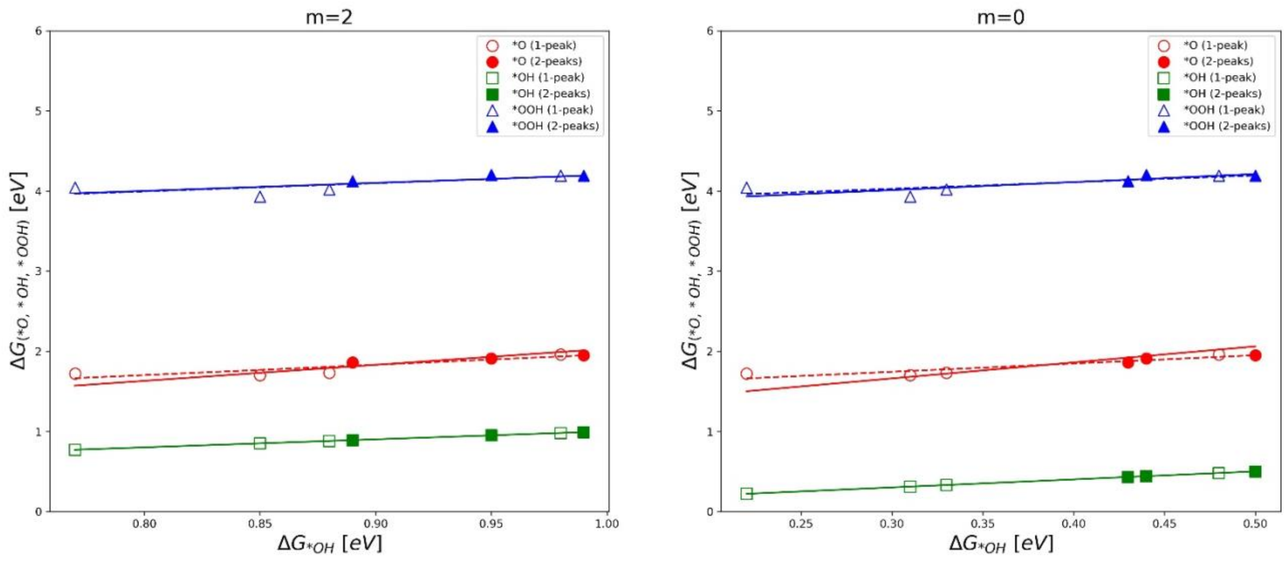

Figure S13: Scaling relationship between the binding energies of *OH, *O and *OOH, for m=2 (a) and m=0 (b). Data points relative to the only showing one CV peak are presented as empty symbols, while full symbols refer to 2-peaks molecules.

Table S2: Scaling relations

Scaling Relation [eV]		OLS （dashed line)		Fix β=1	
		α	β	mean	std
m=2	G(*OH) vs. G(*O)	0.67	1.29	0.03	0.07
	G(*OH) vs. G(*OOH)	3.17	1.03	3.2	0.06
m=0	G(*OH) vs. G(*O)	1.43	1.03	1.06	0.1
	G(*OH) vs. G(*OOH)	3.78	0.83	3.71	0.06

Simulation of X-Ray Absorption Spectra (XAS)

In addition to geometry optimisation we did above we used half core-hole in 1s (hch1s) setup for Fe atom. We calculated the total energy difference of hch1s first core excited state and its ground state as the absolute energy scale. And for spectrum generation we applied Haydock recursion method[12] **(a)** with unit k-points and default parameters suggested by GPAW[13] **(b)**. As we have relatively large molecules, it is computationally infeasible to increase the k-points for a delta Kohn-Sham calculation of the gamma point using the recursion method. This is the reason that we used the total energy difference of hch1s as the scale instead.

Bibliography

1. Shinagawa, T., Garcia-Esparza, A. T. & Takanabe, K. Insight on Tafel slopes from a microkinetic analysis of aqueous electrocatalysis for energy conversion. *Sci. Rep.* **5**, 1–21 (2015).

2. Mefford, J. T., Zhao, Z., Bajdich, M. & Chueh, W. C. Interpreting Tafel behavior of consecutive electrochemical reactions through combined thermodynamic and steady state microkinetic approaches. *Energy Environ. Sci.* **13**, 622–634 (2020).

3. Scott, S. B. *et al.* The low overpotential regime of acidic water oxidation part I: the importance of O2 detection. *Energy Environ. Sci.* 1977–1987 (2022) doi:10.1039/d1ee03914h.

4. Zagal, J. H. & Koper, M. T. M. Reactivity Descriptors for the Activity of Molecular MN4 Catalysts for the Oxygen Reduction Reaction. *Angew. Chemie Int. Ed.* **55**, 14510–14521 (2016).

5. Zagal, J. H. & Koper, M. T. M. Reactivity Descriptors for the Activity of Molecular MN4 Catalysts for the Oxygen Reduction Reaction. *Angew. Chemie - Int. Ed.* **55**, 14510–14521 (2016).

6. Tasca, F. *et al.* Linear versus volcano correlations for the electrocatalytic oxidation of hydrazine on graphite electrodes modified with MN4 macrocyclic complexes. *Electrochim. Acta* **140**, 320–331 (2014).

7. Zúñiga, C. *et al.* Elucidating the mechanism of the oxygen reduction reaction for pyrolyzed Fe-N-C catalysts in basic media. *Electrochem. commun.* **102**, 78–82 (2019).

8. Liu, H. *et al.* A freestanding nanoporous NiCoFeMoMn high-entropy alloy as an efficient electrocatalyst for rapid water splitting. *Chem. Eng. J.* **435**, 134898 (2022).

9. Ashwini, R., Kumar, M. K. P., Rekha, M. Y., Santosh, M. S. & Srivastava, C. Optimization of NiFeCrCoCu high entropy alloy nanoparticle – graphene (HEA-G) composite for the enhanced electrochemical sensitivity towards urea oxidation. *J. Alloys Compd.* **903**, 163846 (2022).

10. Cheng, S. *et al.* Emerging Strategies for CO2 Photoreduction to CH4: From Experimental to Data-Driven Design. *Adv. Energy Mater.* **12**, 2200389 (2022).

11. K. Nørskov, J. *et al.* Origin of the Overpotential for Oxygen Reduction at a Fuel-Cell Cathode. *J. Phys. Chem. B* **108**, 17886–17892 (2004).

12. Haydock, R. The Recursive Solution of the Schrodinger Equation. *Solid State Phys. - Adv. Res. Appl.* **35**, 215–294 (1980).

13. GPAW developers. No Title. *Simulating an XAS spectrum* https://wiki.fysik.dtu.dk/gpaw/tutorialsexercises/opticalresponse/xas/xas.html (2022).
